# Supplementary material for: PI(18:1/18:1) is a SCD1-derived lipokine that limits stress signaling
Source: Nat Commun. 2022 May 27;13:2982. doi: 10.1038/s41467-022-30374-9 (PMC9142606; doi:10.1038/s41467-022-30374-9)
Supplement: Supplementary file 1 — Supplementary Information [file 41467_2022_30374_MOESM1_ESM.pdf]

Supplementary information

PI(18:1/18:1) is a SCD1-derived lipokine that limits stress signaling

Thuermer et al.

Supplementary Figures

Supplementary Fig. 1 | PI and sphingomyelin profiles of fibroblasts heading towards cell death

Supplementary Fig. 2 | Impact of programmed cell death induction on p38 MAPK activation

Supplementary Fig. 3 | Cytotoxic stress depletes cellular MUFAs and modulates LPI ratios

Supplementary Fig. 4 | Role of ACC in MUFA-PI depletion and p38 MAPK activation

Supplementary Fig. 5 | *Scd1* mRNA levels decrease upon cytotoxic treatment

Supplementary Fig. 6 | Regulation of SFA- and MUFA-CoA levels in programmed cell death

Supplementary Fig. 7 | SCD1 inhibition shapes the cellular lipid composition

Supplementary Fig. 8 | Consequences of SCD1 inhibition on PI content and composition

Supplementary Fig. 9 | Silencing of *Scd1* in fibroblasts using siRNA

Supplementary Fig. 10 | CAY10566 activates p38 MAPK through MKK3/6

Supplementary Fig. 11 | Effect of SCD1 inhibition on ER stress, cell viability, and cell death

Supplementary Fig. 12 | Effect of SCD1 inhibition on phospho-tyrosine proteins and their interactome

Supplementary Fig. 13 | Impact of the early cytotoxic decrease of SCD1 expression on stress signaling and cell function

Supplementary Fig. 14 | SCD1 counteracts stress signaling during programmed cell death

Supplementary Fig. 15 | Directionality of MUFA-PI/p38 MAPK regulation

Supplementary Fig. 16 | Role of caspases in cytotoxic SCD1 depletion and stress signaling

Supplementary Fig. 17 | ER stress/UPR and autophagy in *Scd1*-defective mice

Supplementary Fig. 18 | Effect of a defective *Scd1* mutation on phospholipid profiles of murine tissues

Supplementary Fig. 19 | Regulation of major PC and PI species across cytotoxic conditions

Supplementary Fig. 20 | Phospholipid uptake, morphology, and stress marker transcription upon SCD1 inhibition

Supplementary Fig. 21 | SCD1-derived 18:1 suppresses stress signaling

Supplementary Fig. 22 | PI(18:1/18:1) incorporation does not elevate intracellular 18:1 levels

Supplementary Fig. 23 | Impact of SCD1 and SCD1 products on the proteome of stressed cells

Supplementary Fig. 24 | Modulation of enzyme expression in PI biosynthesis and metabolism

Supplementary Fig. 25 | Incorporation of PI(18:1/18:1) into fibroblasts attenuates early cytotoxic stress signaling

Supplementary Fig. 26 | Cytotoxic stress signaling in fibroblasts enriched with PI(18:1/18:1)

Supplementary Fig. 27 | 18:1 decreases death programs in cells surviving VAL exposure

Supplementary Fig. 28 | Uncropped versions of the blots presented in Supplementary Fig. 2a

Supplementary Fig. 39 | Uncropped versions of the blots presented in Supplementary Fig. 2b

Supplementary Fig. 30 | Uncropped versions of the blots presented in Supplementary Fig. 4c, 4e, 9b

Supplementary Fig. 31 | Uncropped versions of the blots presented in Supplementary Fig. 10a

Supplementary Fig. 32 | Uncropped versions of the blots presented in Supplementary Fig. 10b

Supplementary Fig. 33 | Uncropped versions of the blots presented in Supplementary Fig. 10c, 10d

Supplementary Fig. 34 | Uncropped versions of the blots presented in Supplementary Fig. 14a-d

Supplementary Fig. 35 | Uncropped versions of the blots presented in Supplementary Fig. 16a, 16b, 17

Supplementary Fig. 36 | Uncropped versions of the blots presented in Supplementary Fig. 21b

Supplementary Fig. 37 | Uncropped versions of the blots presented in Supplementary Fig. 26a-c

Supplementary Fig. 38 | Gating strategy for Annexin V/propidium iodide staining

Supplementary Tables

Supplementary Table 1 | DEGs related to p38 MAPK activation and stress signaling in starved, *Smed-cct3A* silenced planarians

Supplementary Table 2 | Murine primer sequences used in quantitative RT-PCR experiments

Supplementary Notes

Supplementary Note 1 | PI(18:1/18:1) ratio and p38 MAPK activation across cell lines

Supplementary Note 2 | Detailed descriptions of datasets shown in Fig. 2e

Supplementary Note 3 | MUFA-PI depletion and stress signaling is independent of ACC suppression

Supplementary Note 4 | Functional link between cytotoxic SCD1 depletion and stress signaling

Supplementary Note 5 | p38 MAPK does not shape the phospholipid MUFA composition

Supplementary Note 6 | Caspases are not essential for the early cytotoxic decrease of SCD1

Supplementary Note 7 | Detailed descriptions of datasets shown in Fig. 6

Supplementary Note 8 | Cytotoxic stress impacts the availability of enzymes in PI metabolism

Supplementary Note 9 | Regulation of the stress proteome by SCD1/PI(18:1/18:1)

Supplementary Note 10 | Smed-cct3A knockdown in starved planarians

Supplementary Note 11 | Diversity of mechanisms lowering the cellular PI(18:1/18:1) ratio

Supplementary Note 12 | Kinetics of PI(18:1/18:1) depletion and p38 MAPK activation

Supplementary Note 13 | MUFA-PI candidates with potential stress-reducing activity

Supplementary Figures

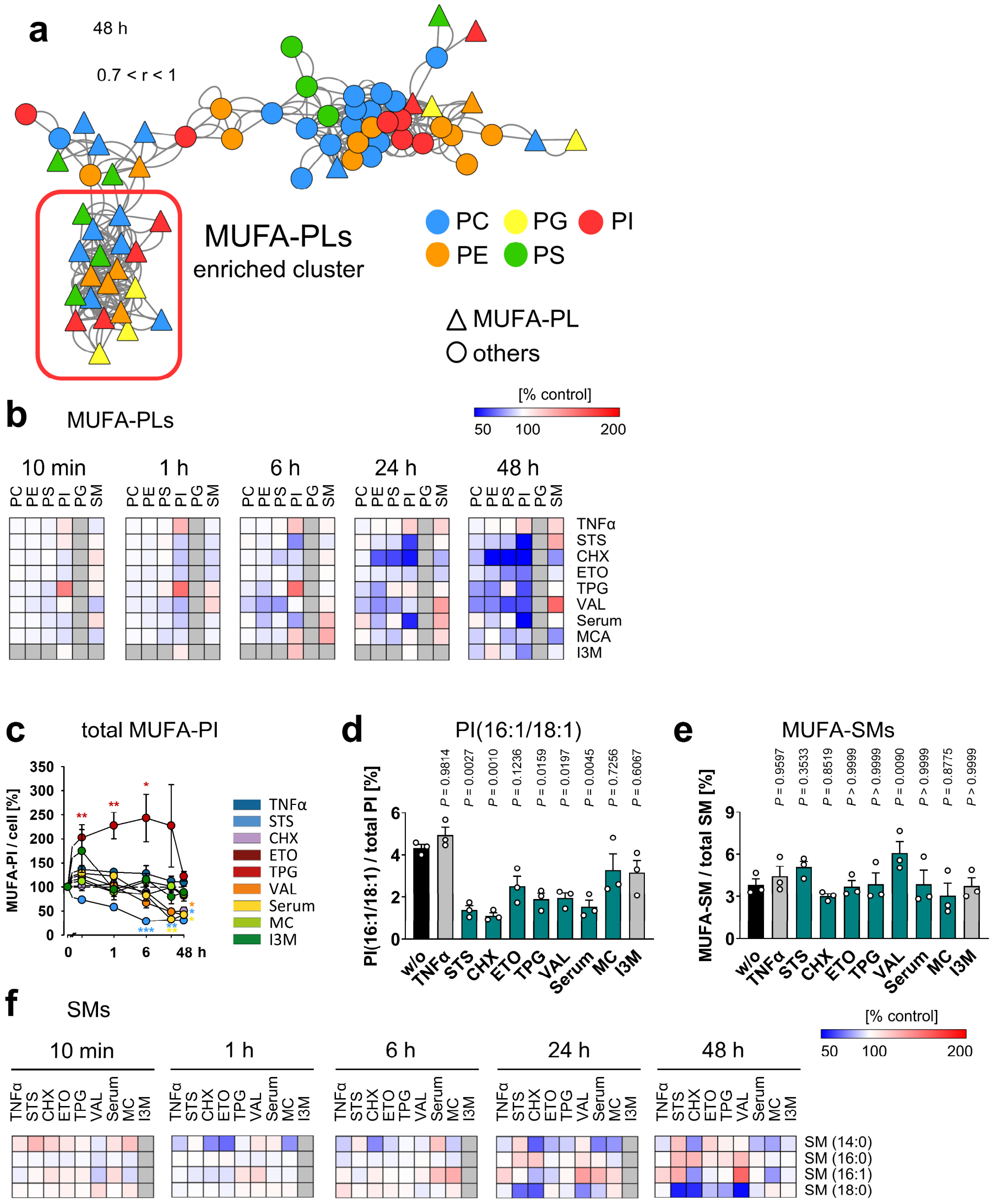

Supplementary Fig. 1 PI and sphingomyelin profiles of fibroblasts heading towards cell death. Cells were cultivated under diverse cytotoxic conditions for 48 h (**a**, **d**, **e**) or as indicated (**b**, **c**, **f**). **a** Network of co-regulated phospholipid (PL) species showing positive lipid-lipid correlations with a correlation factor (r) $\geq$ 0.7. Nodes represent individual lipid species and their colors indicate different headgroups while their shape differentiates between MUFA-containing and -free phospholipids. The network combines data after 48 h treatment and was calculated from mean percentage changes of phospholipid proportions from three independent experiments. **b**, **f** Heatmaps showing the time-dependent changes in phospholipid (PL)-bound MUFAs (**b**) and sphingomyelins (SMs) (**f**). **c** Time-dependent changes of the cellular content of MUFA-PI (TPG LTR P = 0.0018, 0.0030, 0.0165; STS LTR P = 0.0002, 0.0022, 0.0147; VAL P = 0.013; Serum LTR P = 0.0022, 0.0139). **d**, **e** Cellular proportion of PI(16:1/18:1) (**d**) and MUFA-containing SMs (LTR P = 0.99999995, 9999999997, 0.9999999997, 0.9999999995) (**e**). Mean (**a**, **b**, **f**) or mean ± s.e.m. (**c**) and single data (**d**, **e**) from n = 3 independent experiments. *P < 0.05, **P < 0.01, ***P < 0.001 for the respective time point (**c**) or P values given vs. vehicle control (**d**, **e**); repeated measures one-way ANOVA + Tukey HSD post hoc tests.

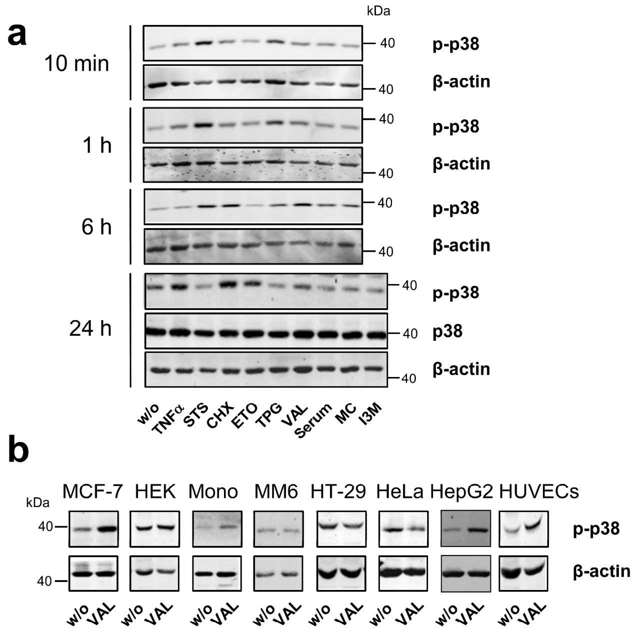

Supplementary Fig. 2 Impact of programmed cell death induction on p38 MAPK activation. **a** Kinetics of p38 MAPK phosphorylation in fibroblasts that were cultivated under diverse cytotoxic conditions. Western blots are representative of three independent experiments. **b** Phosphorylation of p38 MAPK in human cell lines and primary cells that were treated with vehicle or VAL (10 µM) for 48 h. Western blots are representative of two (MM6), three (MCF-7, HEK-293, monocytes, HT-29, and HepG2), four (HeLa and HUVECs) independent experiments.

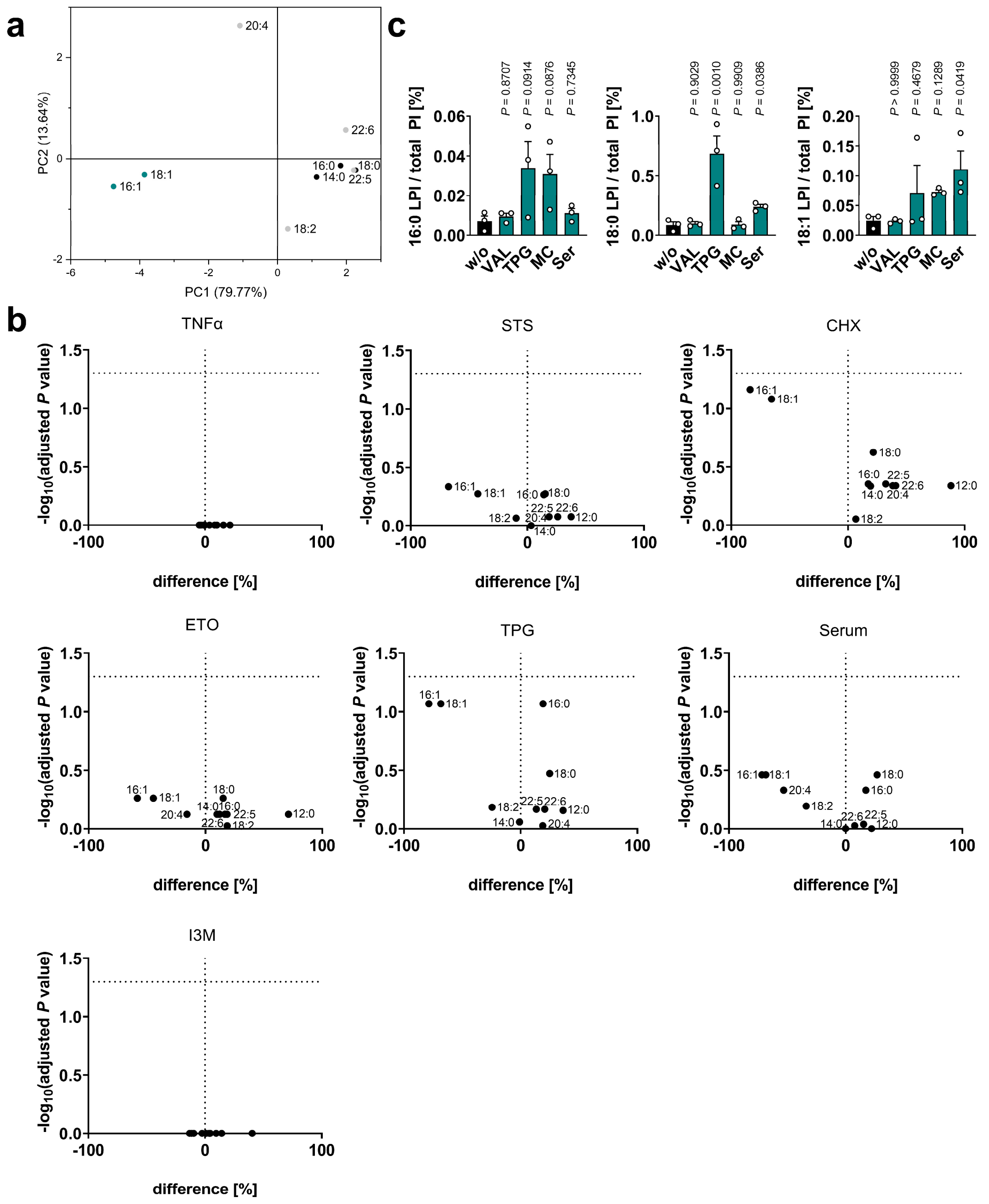

Supplementary Fig. 3 Cytotoxic stress depletes cellular MUFAs and modulates LPI ratios. Fibroblasts were cultivated under diverse cytotoxic conditions for 48 h. **a** Principal component analysis of mean percentage changes in non-esterified fatty acid proportions relative to vehicle control. **b** Volcano plots highlighting free fatty acids, whose levels are substantially modulated under cytotoxic stress conditions. Comparisons of the indicated treatment groups show the mean difference of percentage changes and the negative $\log_{10}$(adjusted *P* value). Adjusted *P* values given vs. vehicle control; two-tailed multiple unpaired student *t*-tests from log data with correction for multiple comparisons using a two-stage linear step-up procedure by Benjamini, Krieger, and Yekutieli (false discovery rate 5%). **c** Cellular proportion of major LPI species (18:0 LPI, MC: $P = 0.99996$). Mean (**a, b**) or mean + s.e.m. and single data (**c**) from $n = 3$ independent experiments. *P* values given vs. vehicle control; repeated measures one-way ANOVA + Tukey HSD post hoc tests of log data.

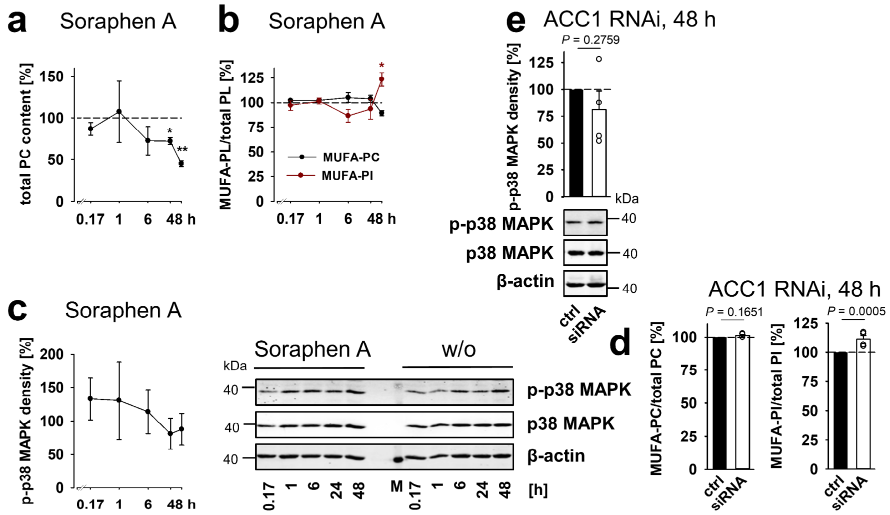

Supplementary Fig. 4 Role of ACC in MUFA-PI depletion and p38 MAPK activation. **a-c** Fibroblasts were treated with the selective ACC inhibitor soraphen A (100 nM) for 10 min to 48 h. **d**, **e** ACC1 was silenced in fibroblasts using siRNA. **a** Cellular content of PC (LTR $P = 0.0272$, 0.0098). **b**, **d** Cellular proportion of MUFA-containing PC and PI (**b**, $P = 0.0499$). **c**, **e** Phosphorylation of p38 MAPK. Western blots are representative of three (**c**) or four (**e**) independent experiments. Mean ± s.e.m. (**a-c**) and single data (**d**, **e**) from $n = 3$ (**a-c**), $n = 4$ (**d**, **e**) independent experiments. $^{*}P < 0.05$, $^{**}P < 0.01$ or P values given vs. vehicle control for each time point (**a-c**) or control siRNA (**d**, **e**); two-tailed paired student *t*-test (**b**, **c**, **d**, **e**) of log data (**a**).

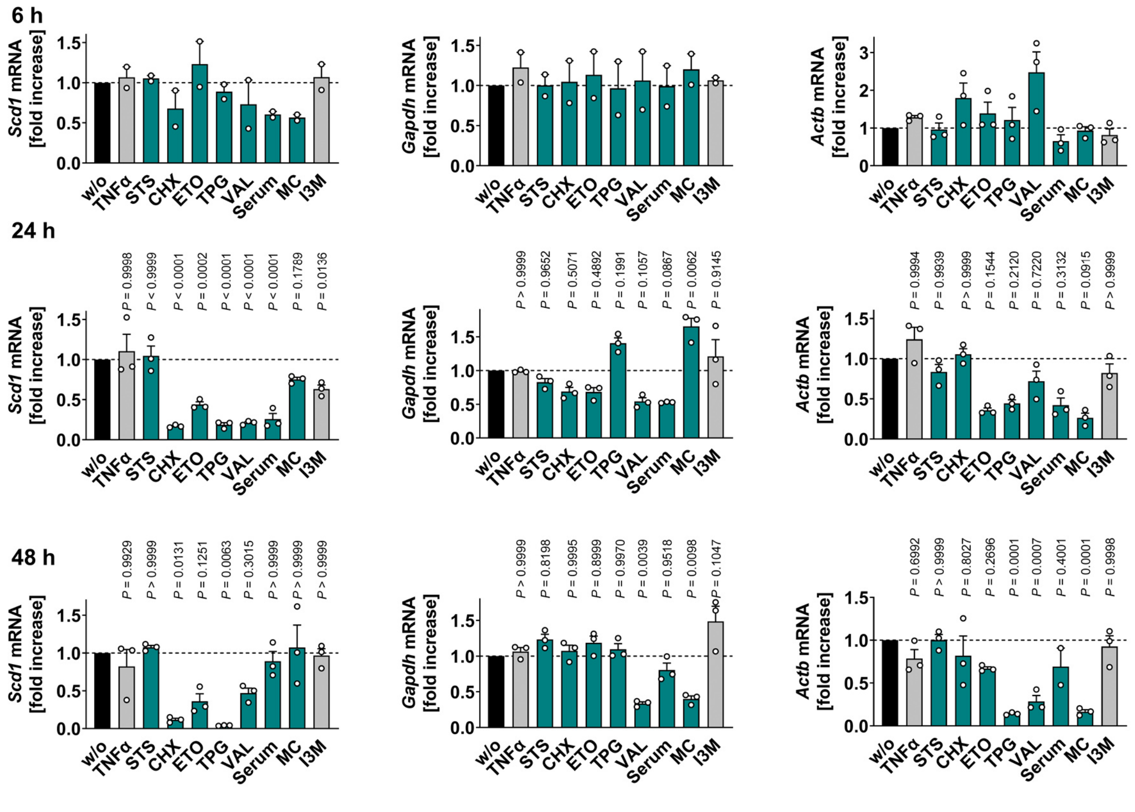

Supplementary Fig. 5 *Scd1* mRNA levels decrease upon cytotoxic treatment. Time-dependent effect on *Scd1*, *Actb*, and *Gapdh* mRNA levels that were normalized to the total amount of cellular RNA. Mean + s.e.m. and single data from n = 3 (except n = 2 for *Scd1* and *Gapdh*, 6h and *Actb*, 48 h, Serum) independent experiments. P values given vs. vehicle control; repeated measures one-way ANOVA or mixed-effects model (REML) (***Actb* 48 h**) + Tukey HSD post hoc tests (24 h LTR P = 0.99999999997, 0.0000008, 0.000001, 0.000002, 0.000003, 0.999999999998, $0.\overline{99}$, 0.99991; 48 h LTR P = 0.999997, 0.99996, 0.99998, 0.999999997, 0.999994, 0.99999999988).

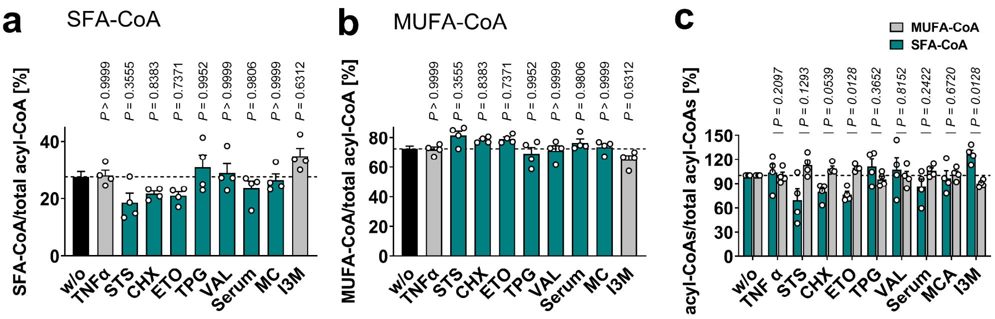

Supplementary Fig. 6 Regulation of SFA- and MUFA-CoA levels in programmed cell death. **a**-**c** Fibroblasts were cultivated under diverse cytotoxic conditions for 48 h, and the cellular proportion of SFA- (LTR P = 0.9999999992, 0.999998, 0.9999992) (**a**) and MUFA-CoA (LTR P = 0.9999999992, 0.999998, 0.9999992) (**b**) was analyzed. **c** Comparison of the percentage changes in SFA- and MUFA-CoAs. Mean + s.e.m. and single data from n = 4 independent experiments. P values given vs. vehicle control; repeated measures one-way ANOVA + Tukey HSD post hoc tests (**a**, **b**) or two-tailed paired student t-test (**c**).

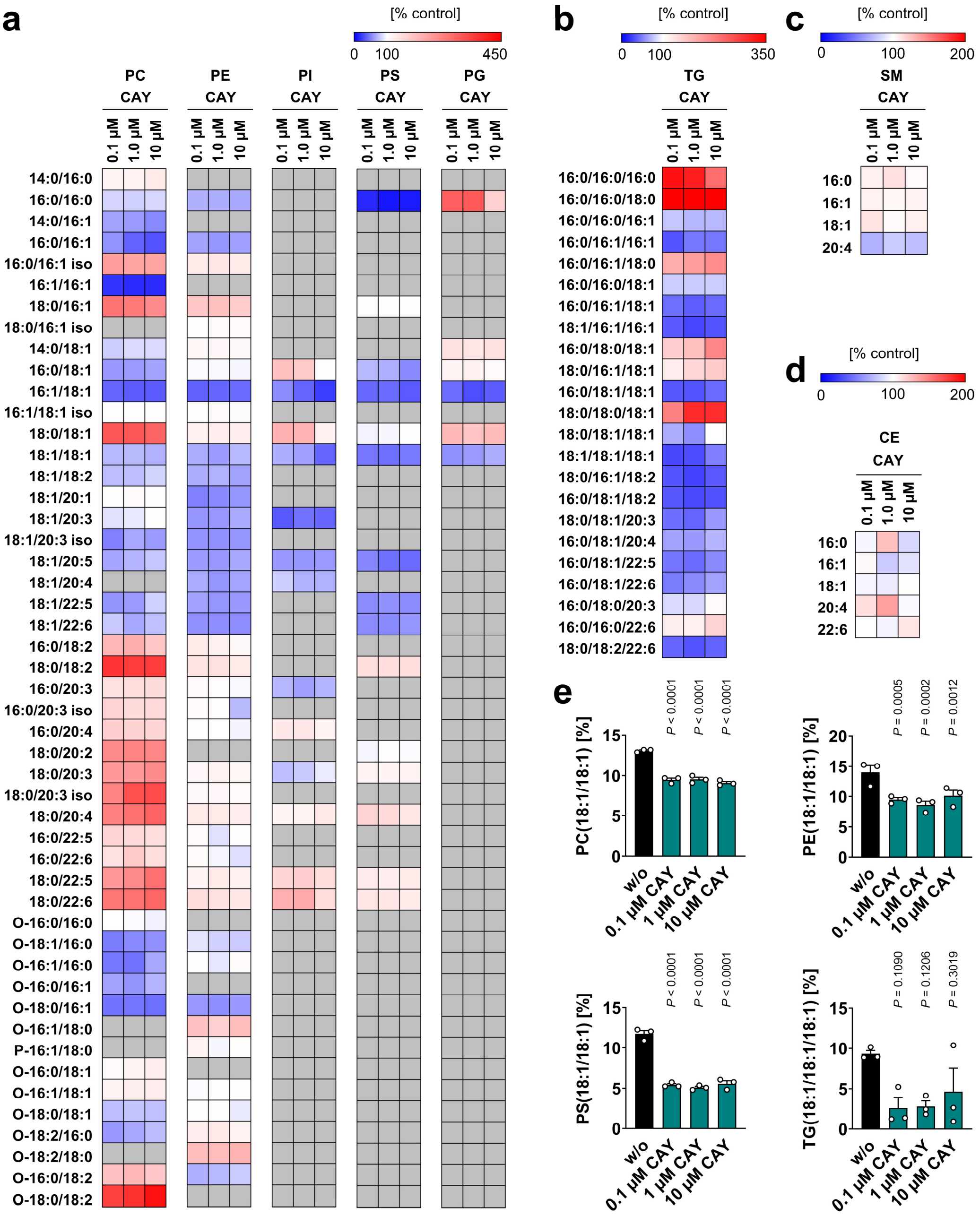

Supplementary Fig. 7 SCD1 inhibition shapes the cellular lipid composition. Fibroblasts were treated with vehicle or CAY10566 (CAY, 0.1, 1, 10 µM) for 48 h. **a-d** Heatmap showing the concentration-dependent changes of the cellular PC, PE, PI, PS, and phosphatidylglycerol (PG) (**a**), triglyceride (TG) (**b**), sphingomyelin (SM) (**c**), and cholesteryl ester (CE) profile (**d**); grey, not determined. Extracts of these dataset have been published before[1]. Data are given as percentage of vehicle control; iso, isomeric species. **e** Cellular proportion of PC(18:1/18:1) (LTR P = 0.00009, 0.00009, 0.00005), PE(18:1/18:1), PS(18:1/18:1) (LTR P = 0.00002, 0.00002, 0.00003), and TG(18:1/18:1/18:1). Mean (**a-d**) or mean + s.e.m. and single data (**e**) from n = 3 independent experiments. P values given vs. vehicle control; repeated measures one-way ANOVA + Tukey HSD post hoc tests.

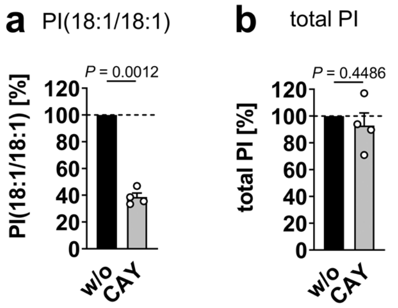

Supplementary Fig. 8 Consequences of SCD1 inhibition on PI content and composition. Fibroblasts were treated with CAY10566 (CAY, 3 µM). **a** Cellular proportion of PI(18.1/18:1). **b** Cellular PI content. Mean + s.e.m. and single data from n = 4 independent experiments. P values vs. vehicle control; two-tailed paired student t-test.

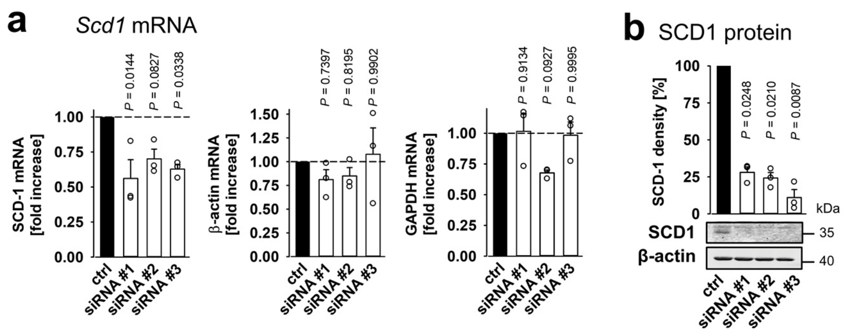

Supplementary Fig. 9 Silencing of *Scd1* in fibroblasts using siRNA. Fibroblasts were transfected with siRNA and incubated for 48 h. **a** *Scd1*, *Actb*, and *Gapdh* mRNA levels were normalized to the total amount of cellular RNA. **b** Protein expression of SCD1. Western blots are representative of three independent experiments. Mean + s.e.m. and single data from *n* = 3 independent experiments. *P* values given vs. control siRNA; ordinary (**a**) or repeated measures (**b**) one-way ANOVA + Tukey HSD post hoc tests.

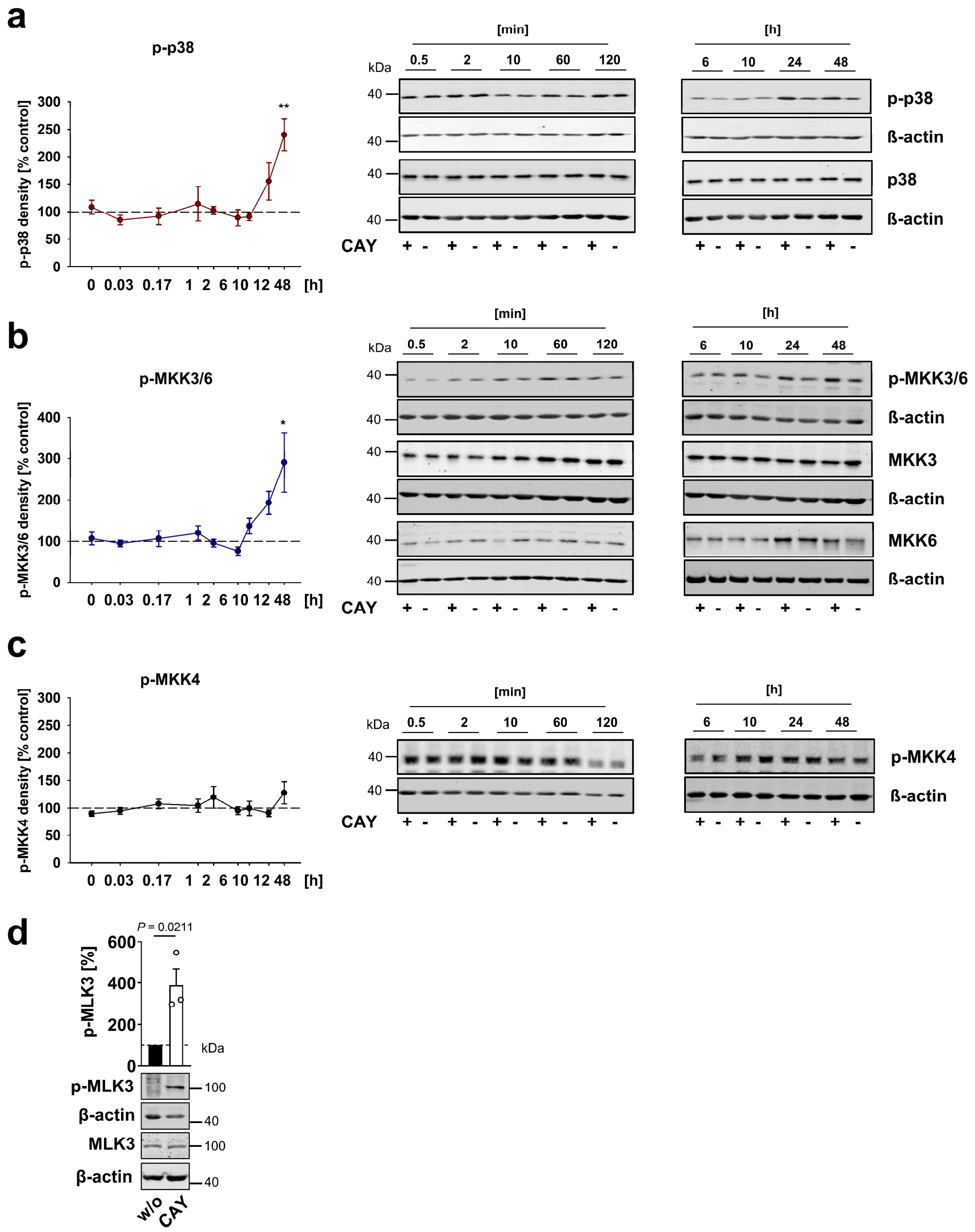

Supplementary Fig. 10 CAY10566 activates p38 MAPK through MKK3/6. Fibroblasts were treated with CAY10566 (CAY, 3 µM). **a-d** Phosphorylation of p38 MAPK (P = 0.0077) (**a**), MKK3/MKK6 (P = 0.0471) (**b**), MKK4 (**c**), and MLK3 (**d**) for 48 h (**d**) or as indicated (**a-c**). Western blots are representative of four (**a**, **b** for 0.5-120 min, **c**) or three (**b** for 6-48 h, **d**) independent experiments. Mean ± s.e.m. from n = 4 (**a**, **b** for 0.5-120 min, **c**), n = 3 (**b** for 6-48 h, **d**) independent experiments. *P < 0.05, **P < 0.01 or P values given vs. vehicle control for each time point; two-tailed paired student t-test (**a-c**) of log data (**d**).

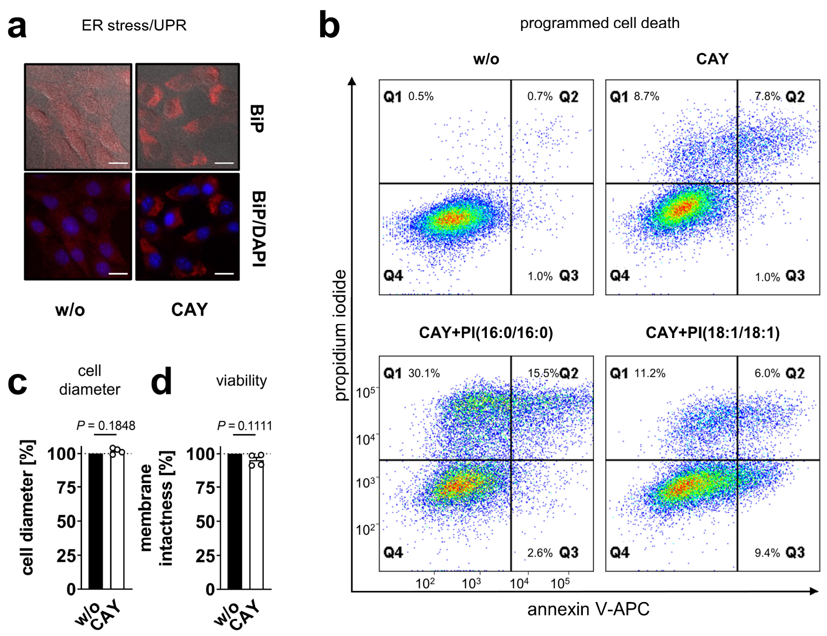

Supplementary Fig. 11 Effect of SCD1 inhibition on ER stress, cell viability, and cell death. Fibroblasts were treated with CAY10566 (CAY, 3 µM), and/or defined phospholipid vesicles (50 µM PI(16:0/16:0) or PI(18:1/18:1)) for 48 h. **a** Subcellular distribution of BiP (red) overlaid with phase contrast images (upper panels) or DAPI staining (blue; lower panels); scale bar, 20 µm. Fluorescence images are representative of four independent experiments. **b** Annexin V and propidium iodide (PI) staining. Cytograms are representative of four independent experiments. Number of cells analyzed: ≥10,000. **c** Cell diameters were analyzed using a Beckmann Vi-CELL Series Cell Counter. **d** Cell membrane integrity determined by trypan blue staining. Mean + s.e.m. and single data (**c, d**) from n = 4 independent experiments. P values given vs. vehicle control; two-tailed paired student t-test.

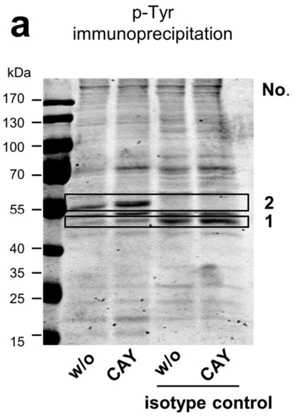

b p-Tyr immunoprecipitates in CAY-treated cells

**No.**	**1**	**2**
**accession**	A2A935.1	Q8VEB4.1
**protein description**	PR domain zinc finger protein 16	Group XV phospholipase A2
**score**	15.34	66.67
**average mass [Da]**	142854.4	47706.5
**protein matched peptides**	1	2
**sequence coverage [%]**	0.71	4.61
**protein amount [ng on column]**	1.433	0.476

Supplementary Fig. 12 Effect of SCD1 inhibition on phospho-tyrosine proteins and their interactome. Fibroblasts were treated with CAY10566 (CAY, 3 µM) for 48 h. **a** SDS-PAGE and Coomassie staining of p-tyrosine immunoprecipitates and the respective isotype controls. Numbers indicate gel sections, in which proteins listed in **b** were detected. **b** Semi-quantitative proteomics reveals tyrosine-phosphorylated proteins in immunoprecipitates that were exclusively detected in CAY-treated fibroblasts but not in the controls.

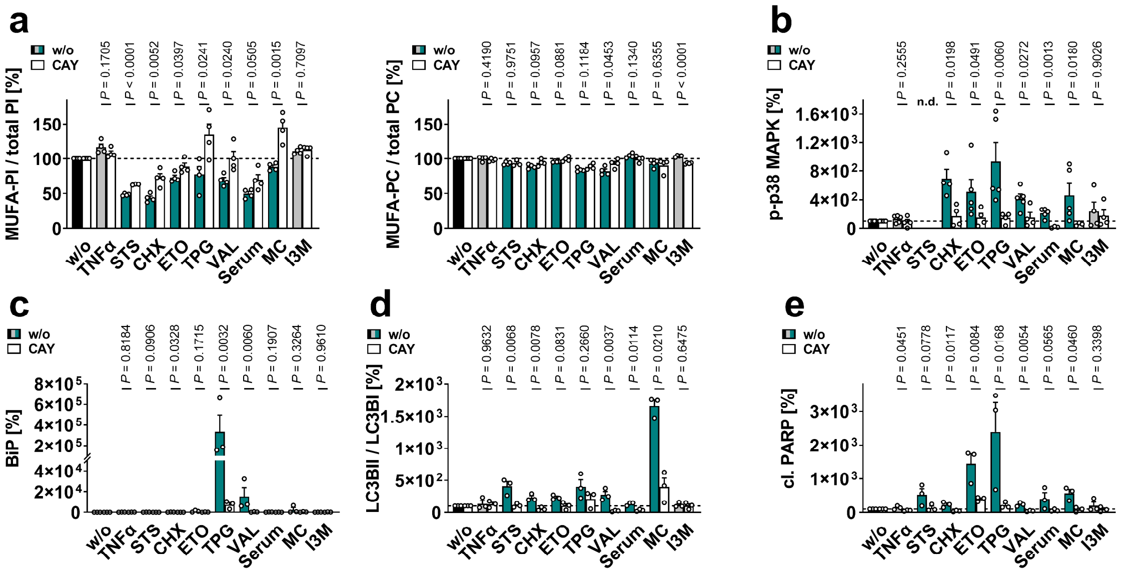

Supplementary Fig. 13 Impact of the early cytotoxic decrease of SCD1 expression on stress signaling and cell function. Fibroblasts were treated with the selective SCD1 inhibitor CAY10566 (CAY, 3 µM) for 48 h, and diverse cytotoxic conditions were applied. To visualize the different responsiveness of cells with active and inactive SCD1 (± CAY) for the cytotoxic agents, vehicle control and CAY-treated cells were set to 100%, respectively. Data for CAY-free experiments are identical to Fig. 1a and d (**a**) or Fig. 2c (**b**). **a** Cellular proportion of PI- and PC-bound MUFAs (LTR $P = 0.00003$, 0.00002). **b** Phosphorylation of p38 MAPK. **c** Protein expression of BiP. **d** Ratio of LC3BII/LC3BI protein levels. **e** PARP cleavage; cl., cleaved. Representative Western blots are shown in Fig. 2c (**b** for w/o), Supplementary Fig. 14a (**b** for CAY) and Supplementary Fig. 14b-d (**c-e**). Mean + s.e.m. and single data from $n = 3$ (**a** STS for CAY, **b** Serum, MC for CAY, **c**, **d**, **e**), $n = 4$ (**a**, **b** for CAY and CHX, I3M for w/o), $n = 5$ (**b** for w/o) independent experiments. Statistical comparisons refer to the responsiveness of cells treated with and without CAY to cytotoxic agents and do not reflect absolute differences. Two-tailed unpaired student *t*-test (**a**) of log data (**b-e**).

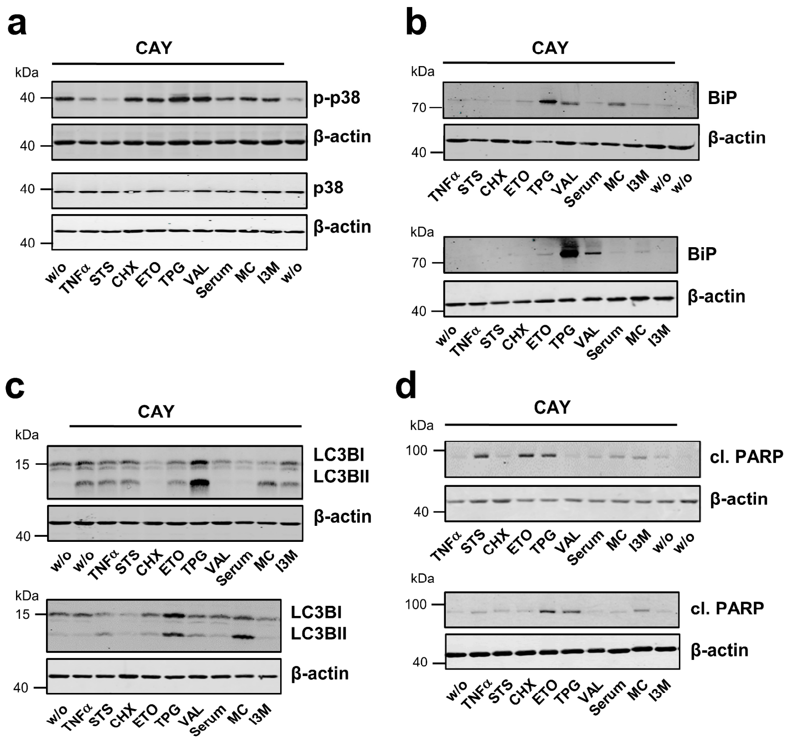

Supplementary Fig. 14 SCD1 counteracts stress signaling during programmed cell death. Fibroblasts were treated with CAY10566 (CAY, 3 μM) for 48 h, and diverse cytotoxic conditions were applied. **a** Phosphorylation of p38 MAPK. **b** Protein expression of BiP. **c** Protein levels of LC3BI and LC3BII. **d** PARP cleavage; cl., cleaved. Western blots are representative of three (**b**, **c**, **d** for cytotoxic conditions + CAY), four (**a**), or six (**d** for cytotoxic conditions) independent experiments.

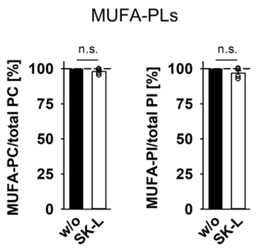

Supplementary Fig. 15 Directionality of MUFA-PI/p38 MAPK regulation. Fibroblasts were treated with the selective p38 MAPK inhibitor Skepinone L (SK-L, 0.3 µM) for 48 h, and the percentage change in cellular proportions of MUFA-bound PC and PI were analyzed. Mean + s.e.m. and single data from *n* = 4 independent experiments; two-tailed paired student *t*-test.

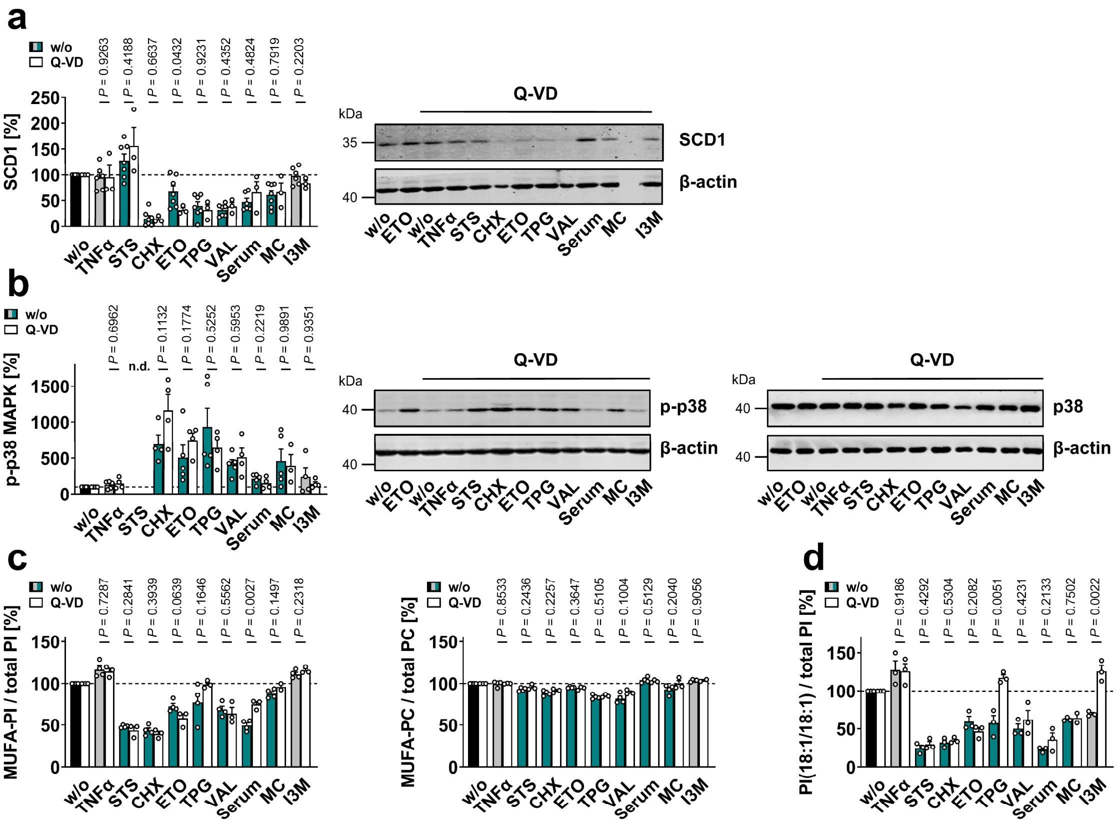

Supplementary Fig. 16 Role of caspases in cytotoxic SCD1 depletion and stress signaling. Fibroblasts were treated with the pan-caspase inhibitor Q-VD-OPh (Q-VD; 20 µM) for 48 h, and diverse cytotoxic conditions were applied. To visualize the different responsiveness of cells with or without caspase inhibition for the cytotoxic agents, vehicle control and Q-VD-treated cells were set to 100%, respectively. Data for Q-VD-free experiments (w/o) are identical to Fig. 3e (**a**), Fig. 2c (**b**) or Fig. 1a, d (**c**). **a** Protein expression of SCD1. **b** Phosphorylation of p38-MAPK. Western blots for Q-VD are representative of three (**a**) or four (**b**) independent experiments. Representative Western blots for Q-VD-free experiments are shown in Fig. 3e (**a**) and Fig. 2c (**b**). **c**, **d** Changes in the cellular proportion of MUFA-containing PI and PC (**c**) and PI(18:1/18:1) (**d**). Mean + s.e.m. and single data from $n = 2$ (**c**, MC for Q-VD (PI) and I3M for Q-VD (PC), **d**, MC for Q-VD), $n = 3$ (**a**, Q-VD, **b**, MC for Q-VD, **c**, for Q-VD, **d**), $n = 4$ (**b** for Q-VD and CHX, I3M for w/o, **c** for w/o), $n = 5$ (**b** for w/o), $n = 6$ (**a** TNFα, Serum, I3M for w/o), $n = 7$ (**a**, for w/o) independent experiments. Statistical comparisons refer to the responsiveness of cells treated with and without Q-VD to cytotoxic agents and do not reflect absolute differences. Two-tailed unpaired student *t*-test (**c**, **d**) of log data (**a**, **b**).

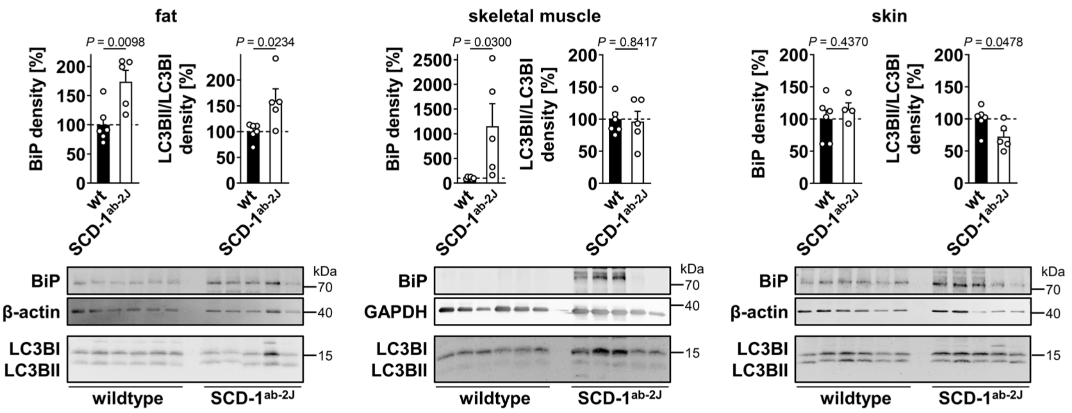

Supplementary Fig. 17 ER stress/UPR and autophagy in *Scd1*-defective mice. White abdominal fat, hind leg skeletal muscle, and skin were obtained from wildtype mice (wt) and mice homozygous for the *Scd1*$^{\text{ab-2J}}$ allele (*Scd1*$^{\text{ab-2J}}$). Protein expression of BiP and the ratio of LC3BII/LC3BI protein levels are shown. BiP signals were normalized to either β-actin (fat, skin) or GAPDH (skeletal muscle) protein levels. Mean + s.e.m. and single data from $n = 6$ (wt), $n = 5$ (*Scd1*$^{\text{ab-2J}}$), $n = 4$ (BiP for skin, *Scd1*$^{\text{ab-2J}}$) mice. *P* values given vs. wildtype; two-tailed unpaired student *t*-test.

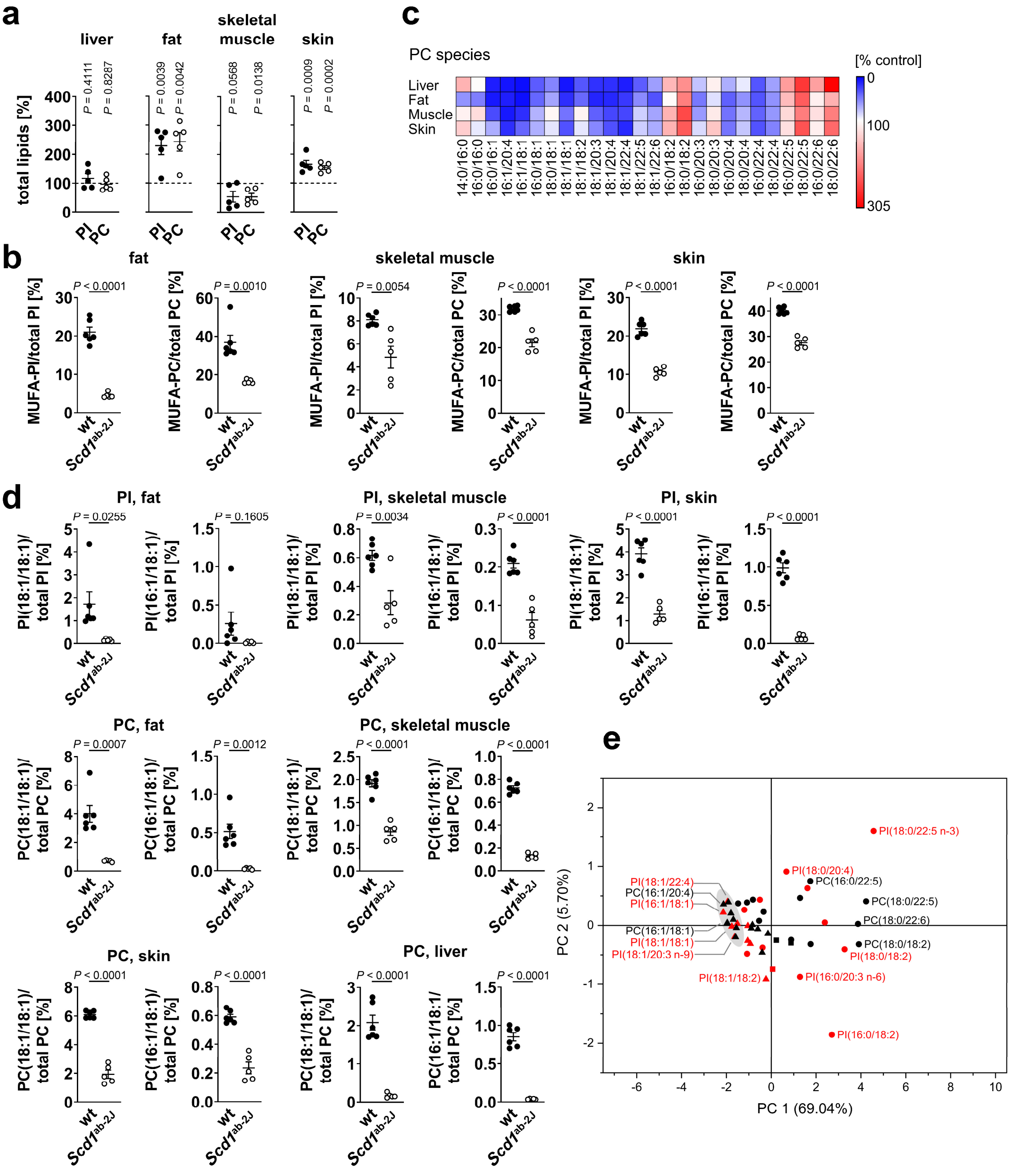

Supplementary Fig. 18 Effect of a defective *Scd1* mutation on phospholipid profiles of murine tissues. Liver, white abdominal fat, hind leg skeletal muscle, and skin were obtained from wildtype (wt) mice and mice homozygous for the *Scd1*$^{\text{ab-2J}}$ allele (*Scd1*$^{\text{ab-2J}}$). **a** Tissue content of PI and PC. **b** Proportion of MUFAs in PI and PC; MUFAs: 16:1, 18:1 (LTR *P* = 0.000001, 0.00001, 0.0000006, 0.0000008). **c** Heatmap showing the changes in the proportion of PC species from liver, fat, skeletal muscle, and skin from *Scd1*$^{\text{ab-2J}}$ mice as compared to wt mice. **d** Proportion of PI(18:1/18:1), PI(16:1/18:1), PC(18:1/18:1), and PC(16:1/18:1) (LTR *P* = 0.00007, 0.00002, 0.0000005, 0.00001, 0.000000002, 0.0000001, 0.00001, 0.0000096, 0.0000002). **e** Principal component analysis of PC and PI species from liver, fat, skeletal muscle and skin from *Scd1*$^{\text{ab-2J}}$ mice based on mean percentages of wt control. Mean (**c**) or mean + s.e.m. and single data (**a**, **b**, **d**) from *n* = 6 (wt) and *n* = 5 (*Scd1*$^{\text{ab-2J}}$) mice. *P* values given vs. wildtype; two-tailed unpaired student *t*-test.

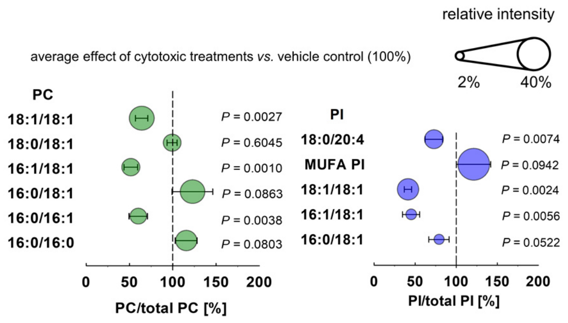

Supplementary Fig. 19 Regulation of major PC and PI species across cytotoxic conditions. Fibroblasts were cultivated under diverse cytotoxic conditions for 48 h. Effects of the cytotoxic settings described in Fig. 1 on the cellular proportion were averaged for each of the major PC and PI species shown, and the changes are compared to vehicle control (100%). Mean ± lower and upper endpoint of the confidence interval (95%) from $n = 4$ independent experiments. Dot sizes indicate the cellular proportion given as relative intensities. *P* values given vs. vehicle control; two-tailed paired student *t*-test.

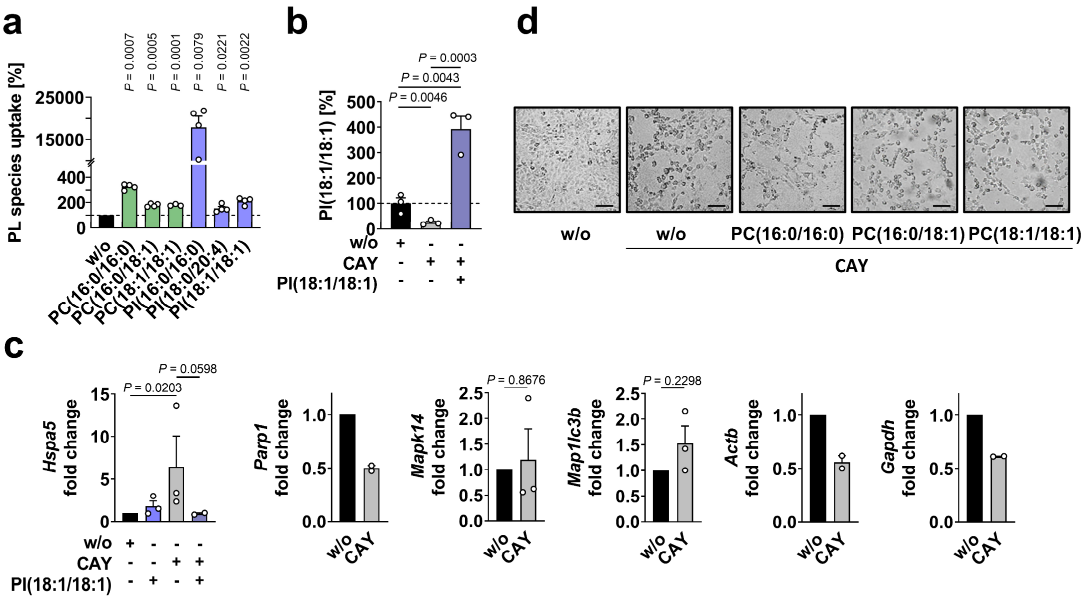

Supplementary Fig. 20 Phospholipid uptake, morphology, and stress marker transcription upon SCD1 inhibition. Fibroblasts were treated with vehicle, CAY10566 (CAY, 3 µM) and/or defined phospholipid vesicles (50 µM) for 48 h. **a** Cellular uptake of PC and PI species. **b** Cellular content of PI(18:1/18.1). **c** mRNA levels of *Bip* (*Hspa5*), *Parp1*, *p38 MAPK* (*Mapk14*), *LC3B* (*Map1lc3b*), *β-actin* (*Actb*), and *Gapdh* normalized to the total amount of cellular RNA and compared to vehicle control. Mean + s.e.m. and single data from n = 2 (**c**, CAY + PI(18:1/18:1) for *Hspa5*, CAY for *Parp1, Actb, Gapdh*), *n* = 3 (**a**, for PC(18:1/18:1), **b, c**), *n* = 4 (**a**)independent experiments. *P* values given vs. vehicle control or as indicated; two-tailed paired student *t*-test (**a, c**) or repeated measures one-way ANOVA (**b**) or mixed-effects model (REML) (**c**, for *Hspa5*) + Tukey HSD post hoc tests of log data. **d** Fibroblast morphology; scale bar, 100 µm. Phase contrast images are representative of three independent experiments. Significant outliers (**c**) were removed (Grubbs´ test, p < 0.05).

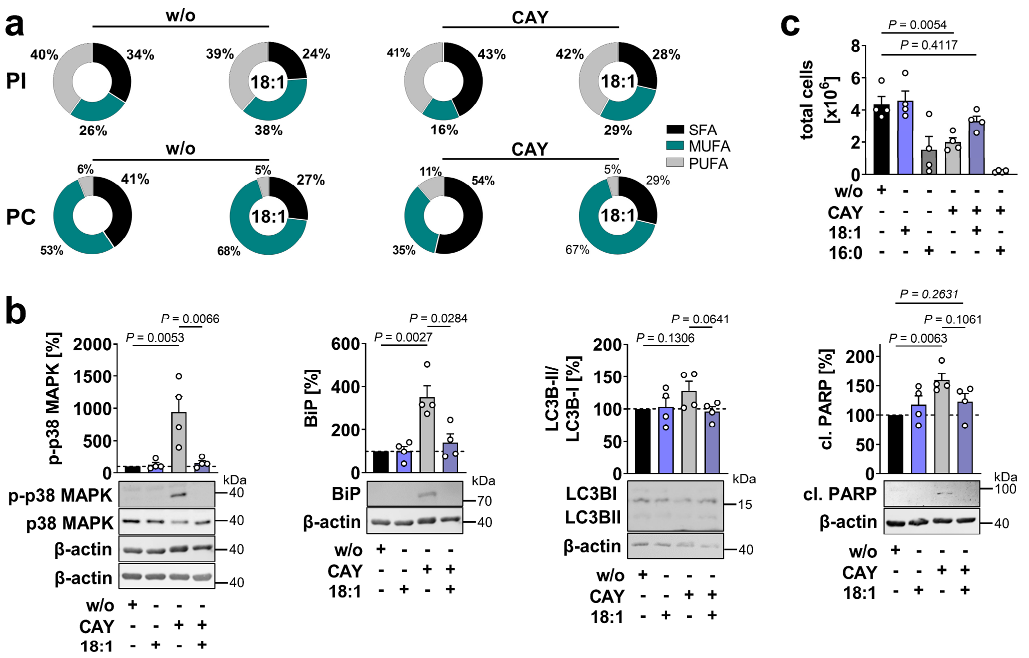

Supplementary Fig. 21 SCD1-derived 18:1 suppresses stress signaling. Fibroblasts were treated with vehicle, CAY10566 (CAY, 3 µM), and/or 16:0 or 18:1 (100 µM) for 48 h. **a** Cellular proportion SFA, MUFA, and PUFA within PI and PC. SFAs: 14:0, 16:0, 18:0; MUFAs: 16:1, 18:1; PUFAs: 18:2, 20:3, 20:4, 22:4, 22:5, 22:6. **b** Phosphorylation of p38 MAPK, protein expression of BiP, ratio of LC3BII/LC3BI protein levels, and PARP cleavage; cl., cleaved. Western Blots are representative of four independent experiments. **c** Cell numbers. Mean (**a**) or mean + s.e.m. and single data (**b**, **c**) from $n = 4$ independent experiments. Two-tailed unpaired student t-test of log data (**b**) or repeated measures one-way ANOVA + Tukey HSD post hoc tests (**c**).

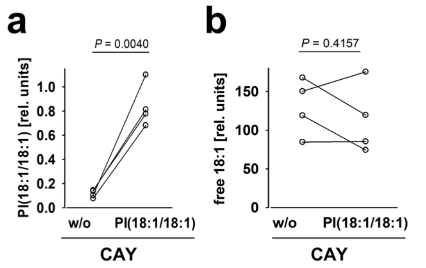

Supplementary Fig. 22 PI(18:1/18:1) incorporation does not elevate intracellular 18:1 levels. Fibroblasts were treated with CAY10566 (CAY, 3µM) and/or PI(18:1/18:1) vesicles (50 µM) for 48 h. **a**, **b** Cellular content of PI(18:1/18:1) (**a**) and 18:1 (**b**). Paired data from *n* = 4 independent experiments. *P* values given vs. vehicle control; two-tailed paired student *t*-test.

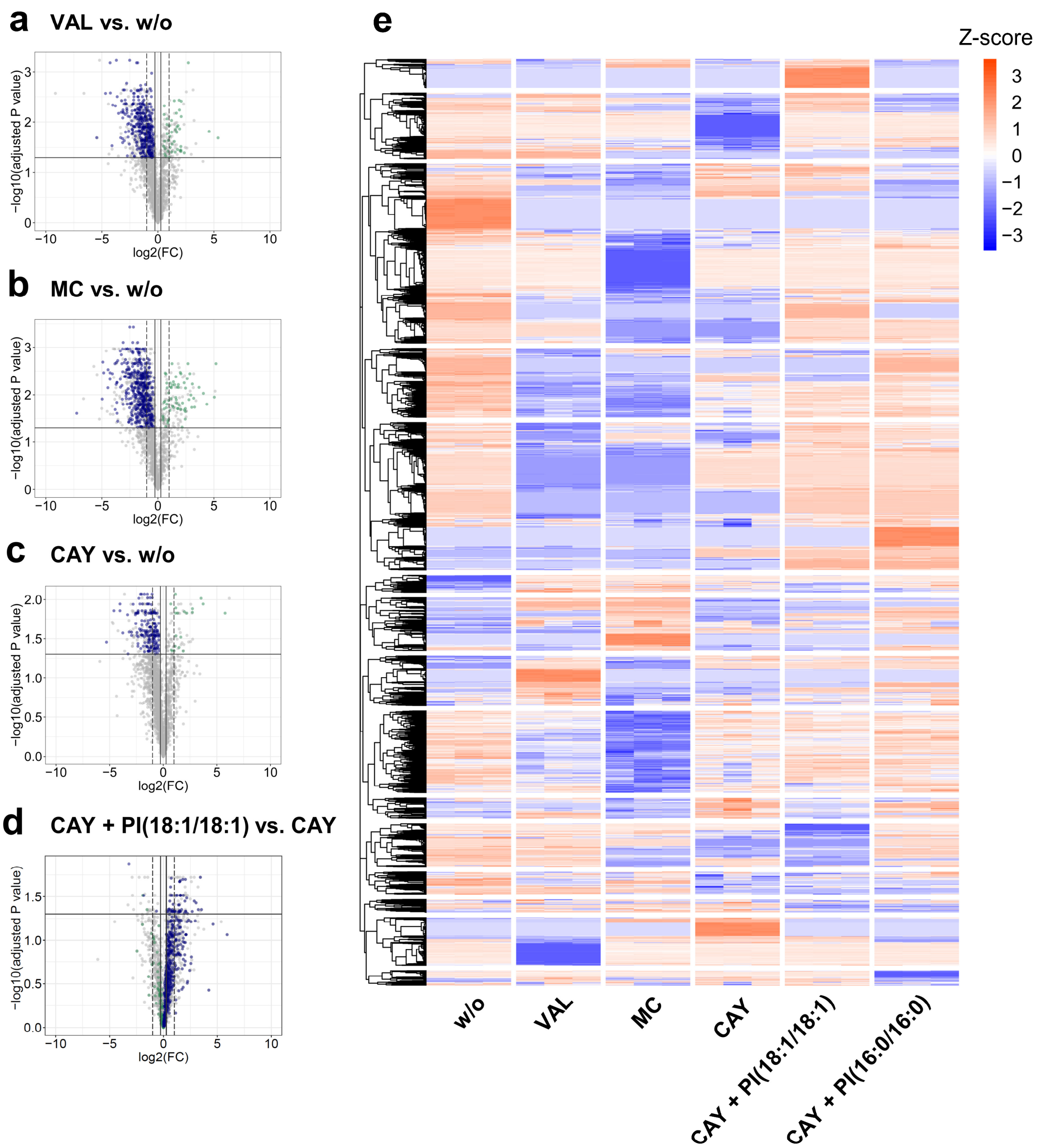

Supplementary Fig. 23 Impact of SCD1 and SCD1 products on the proteome of stressed cells. Fibroblasts were treated with vehicle, VAL (10 µM), MC (10 µM), CAY10566 (CAY, 3 µM) in presence or absence of either PI(18:1/18:1 (50 µM) or PI(16:0/16:0) for 48 h. **a-d** Volcano plots, where the log2 fold change (log2(FC)) is plotted against the negative log10 of adjusted *P* value, highlight proteins that are regulated by VAL (**a**), MC (**b**), or CAY (**c**) relative to vehicle control or by PI(18:1/18:1) supplementation in CAY-treated cells (**d**). For statistical analysis, an unpaired, two-tailed Welch *t*-test was used and adjusted *P*-values (correction for multiple comparisons) were calculated using Benjamini-Hochberg correction. Proteins that are consistently down- (blue) or upregulated (green) (≥ 20%) by all three stressors (VAL, MC, CAY) *versus* vehicle control are indicated by color. **e** Heatmap of proteins identified in the independent experiments of vehicle control, VAL-, MC-, CAY-, CAY+PI(18:1/18:1)- and CAY+PI(18:1/18:1)-treated cells. Protein intensities were log2 transformed and are colored based on their z-score ranging from -3 to 3.

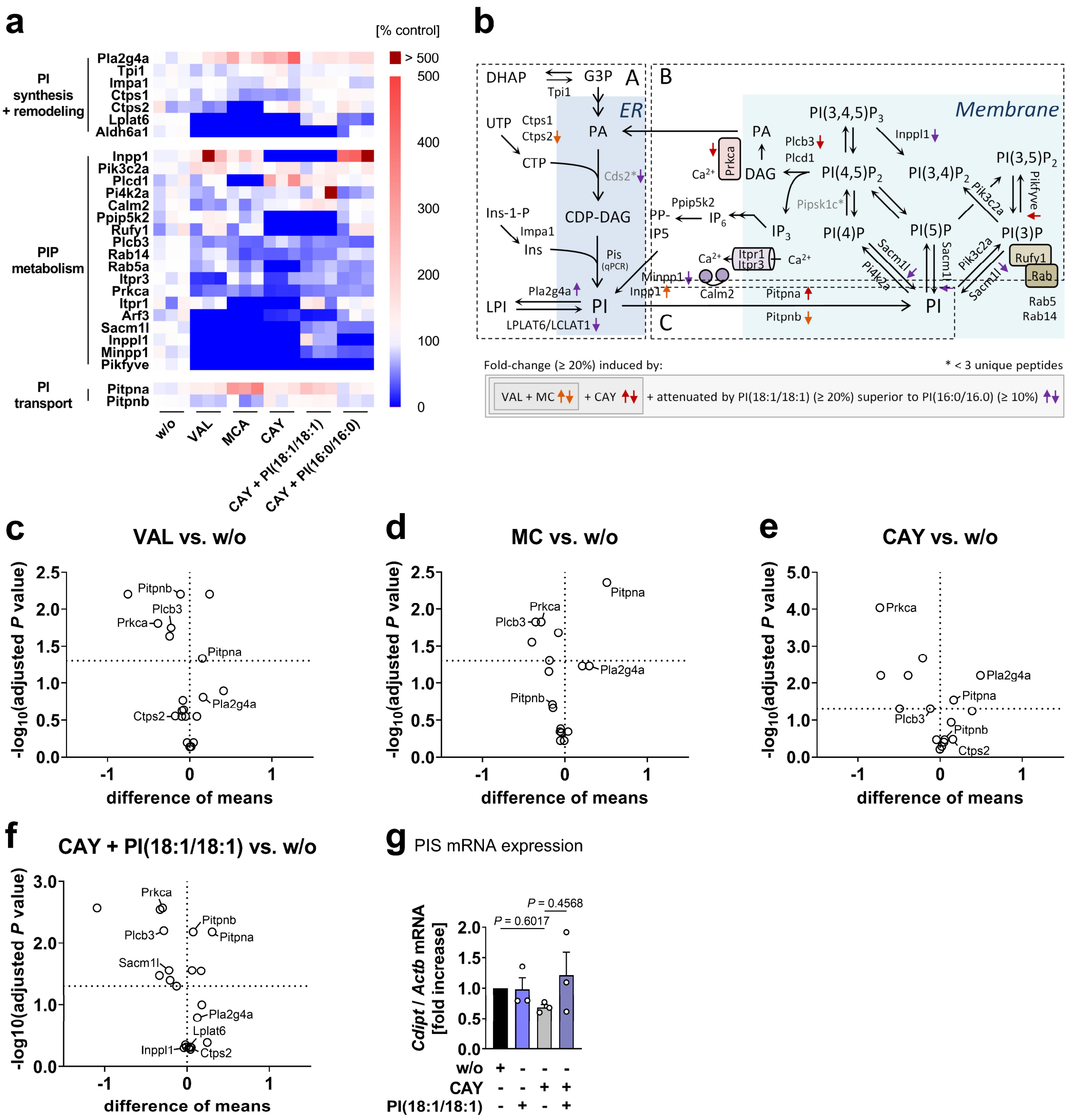

Supplementary Fig. 24 Modulation of enzyme expression in PI biosynthesis and metabolism. Fibroblasts were treated with vehicle, VAL (10 µM), MC (10 µM), or CAY10566 (CAY, 3 µM), CAY (3 µM) plus PI(18:1/18:1) (50 µM), or CAY (3 µM) plus PI(16:0/16:0) (50 µM) for 48 h. a Heatmap showing relative changes in the cellular level of proteins involved in PI biosynthesis and metabolism. Data of independent experiments (n = 3) were calculated as percentage of vehicle control. b Schematic overview of PI biosynthesis (A), PI transport between ER and other cellular membranes (B), and PIP metabolism (C). Proteins detected by quantitative proteomics are labeled. Arrows indicate up- or downregulation of cellular protein levels by the treatments as indicated. De novo PI biosynthesis starts at the endoplasmic reticulum (ER) from glycerol-3-phosphate (G3P), which is converted into phosphatidic acid (PA) and coupled to cytosine triphosphate (CTP) by phosphatidate cytidylyltransferase (Cds) to yield CDP-diacylglycerol (CDP-DAG). Subsequent coupling of CDP-DAG and myo-inositol (Ins) by phosphatidylinositol synthase (Pis) produces PI. The *sn*-2 fatty acid of PI is continuously exchanged by the concerted activities of phospholipases (Pla)A_2 and lysophospholipid acyltransferases (Lplat) (remodeling pathway). PI is distributed from the ER to other cellular membrane pools, among others by phosphatidylinositol transfer proteins (PITPs). Position-specific kinases phosphorylate PI to produce PI-(3/4/5)-phosphates (PI(3/4/5)P2) as well as di- and triphosphates with important functions in vesicle transport, autophagy, cytoskeleton organization, and receptor signaling. Phosphatases terminate the signal by dephosphorylating PIPs.

Moreover, PI(4,5)P2 is cleaved by phospholipase C (Plc) to DAG and inositol-1,3,5-trisphosphate (IP3), which results in the activation of ER-located IP3 receptors (Itpr), Ca^{2+}-influx into the cytosol, and activation of protein kinase C (Prkc). c-f Volcano plots highlighting proteins that are regulated by VAL, MC, or CAY relative to vehicle control or by PI(18:1/18:1) supplementation in CAY-treated cells. Comparisons of the indicated treatment groups show the difference of mean absolute intensities of log10 data and the negative log10(adjusted P value). Adjusted P values given vs. vehicle control; two-tailed multiple unpaired student t-tests from log data with correction for multiple comparisons using a two-stage linear step-up procedure by Benjamini, Krieger, and Yekutieli (false discovery rate 5%). g mRNA level of Cdipt (Pis) normalized to Actb. Single data (a), mean (c-f) or mean + s.e.m. and single data (g) from n = 3 independent experiments. Repeated measures one-way ANOVA + Tukey HSD post hoc tests (g).

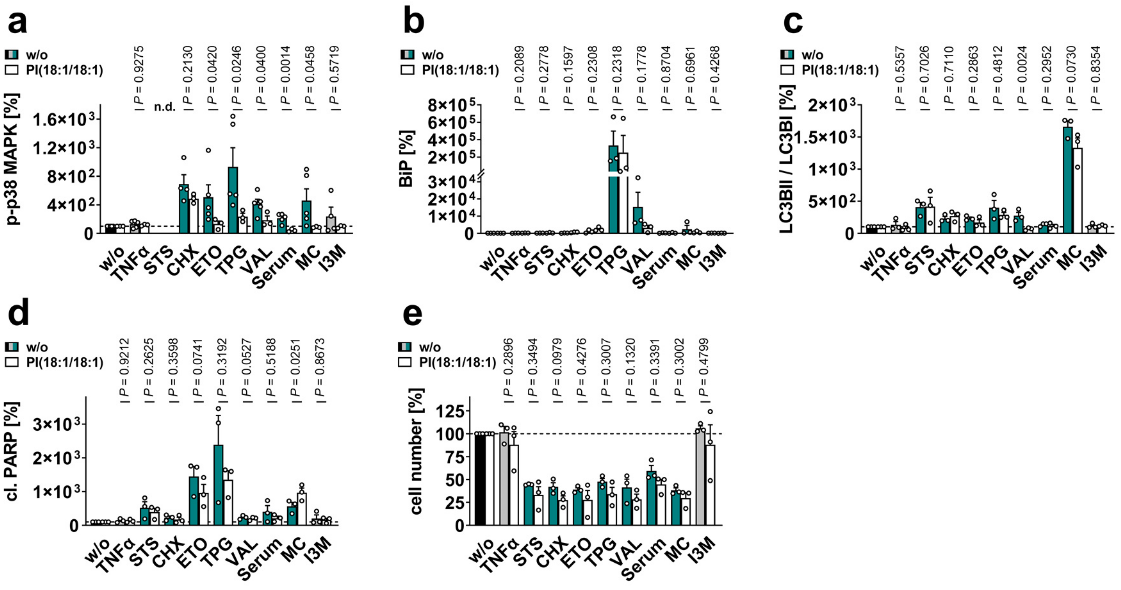

Supplementary Fig. 25 Incorporation of PI(18:1/18:1) into fibroblasts attenuates early cytotoxic stress signaling. Fibroblasts were supplemented with PI(18:1/18:1) vesicles (50 µM) for 48 h, and diverse cytotoxic conditions were applied. To visualize the different responsiveness of cells with or without PI(18:1/18:1) supplementation for the cytotoxic agents, vehicle control and PI(18:1/18:1)-treated cells were set to 100%, respectively. Data for w/o are identical to Fig. 2c (**a**) or Supplementary Fig. 13c-e (**b-d**). **a** Phosphorylation of p38 MAPK. **b** Protein expression of BiP. **c** Ratio of LC3BII/LC3BI protein levels. **d** PARP cleavage; cl., cleaved. Representative Western blots are shown in Fig. 2c and Supplementary Fig. 14 and 26. **e** Cell numbers. Mean + s.e.m. and single data from n = 3 (**a** for PI(18:1/18:1), **b-e**), n = 4 (**a**, CHX, I3M for w/o), n = 5 (**a** for w/o) independent experiments. Statistical comparisons refer to the responsiveness of cells treated with and without PI(18:1/18:1) to cytotoxic agents and do not reflect absolute differences. Two-tailed unpaired (**a**) or paired (**b-e**) student t-test of log data.

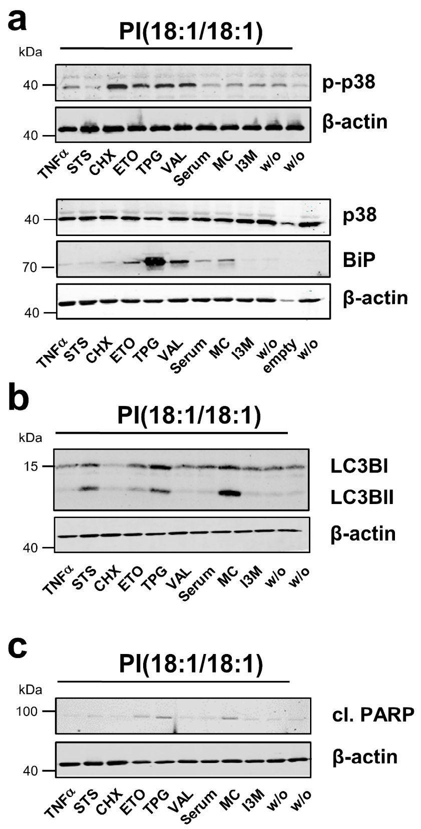

Supplementary Fig. 26 Cytotoxic stress signaling in fibroblasts enriched with PI(18:1/18:1). Fibroblasts were supplemented with PI(18:1/18:1) vesicles (50 µM) for 48 h, and diverse cytotoxic conditions were applied. **a** Phosphorylation and protein expression of p38 MAPK and BiP. **b** Protein levels of LC3BI and LC3BII. **c** PARP cleavage; cl., cleaved. Western blots are representative of three independent experiments.

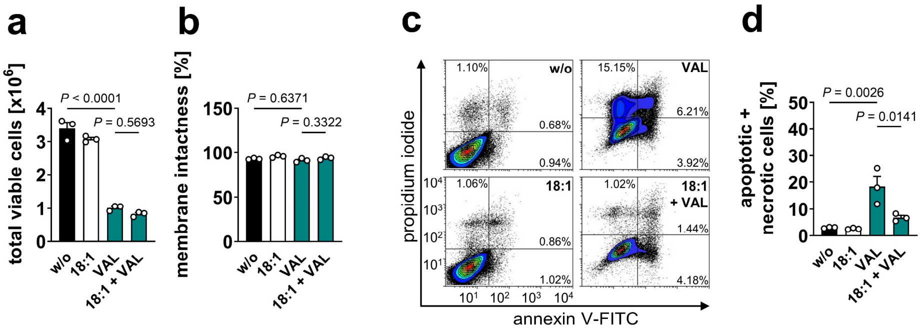

Supplementary Fig. 27 18:1 decreases death programs in cells surviving VAL exposure. Fibroblasts were treated with vehicle or VAL (10 µM) in presence or absence of 18:1 (100 µM) for 48 h. **a**, **b** Viable cell number ($P = 0.0000007$) (**a**) and membrane intactness (**b**). **c**, **d** Annexin V and propidium iodide (PI) staining of cells that remain attached after 48 h treatment and rinsing. **c** Cytograms are representative of three independent experiments. For each condition, 200,000 cells were analyzed. Gating was adjusted according to the double-negative cell population. **d** Apoptotic/necrotic cells comprise apoptotic, late apoptotic, and necrotic cells, as defined for Fig. 4i. Mean + s.e.m. and single data from $n = 3$ independent experiments. P values given as indicated; ordinary one-way ANOVA + Tukey HSD post hoc tests.

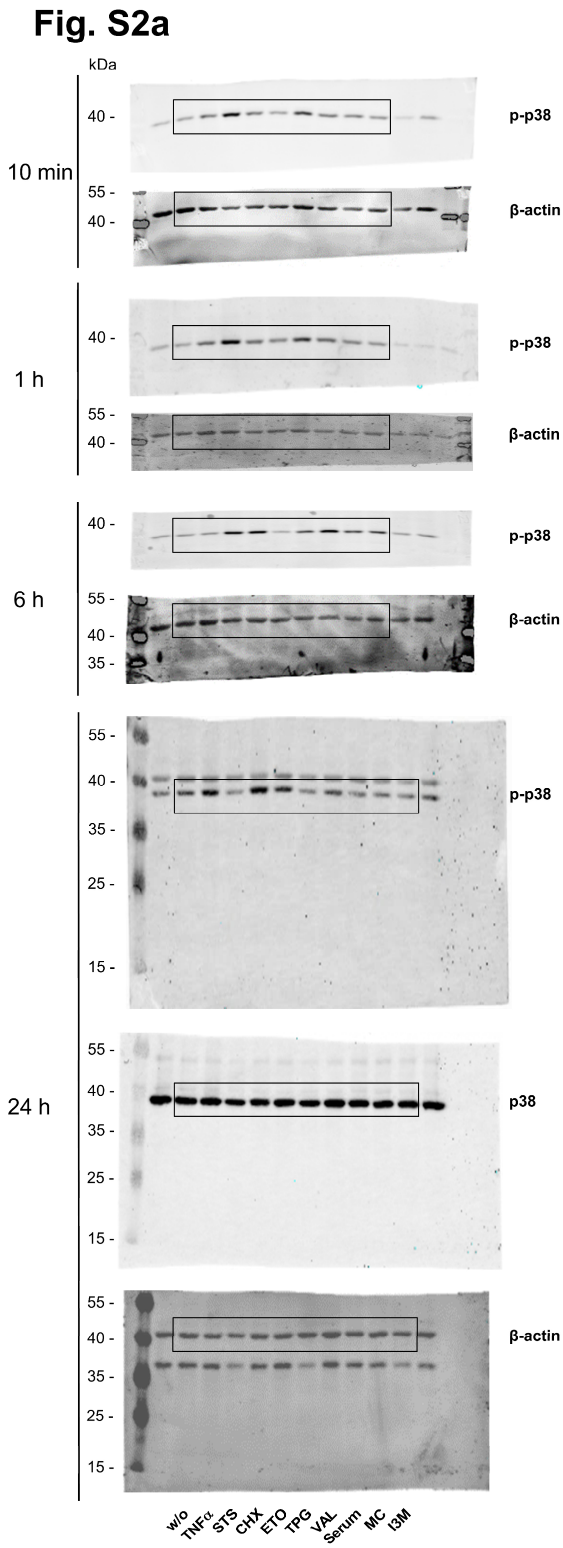

Supplementary Fig. 28 Uncropped versions of the blots presented in Supplementary Fig. 2a.

Fig. S2b

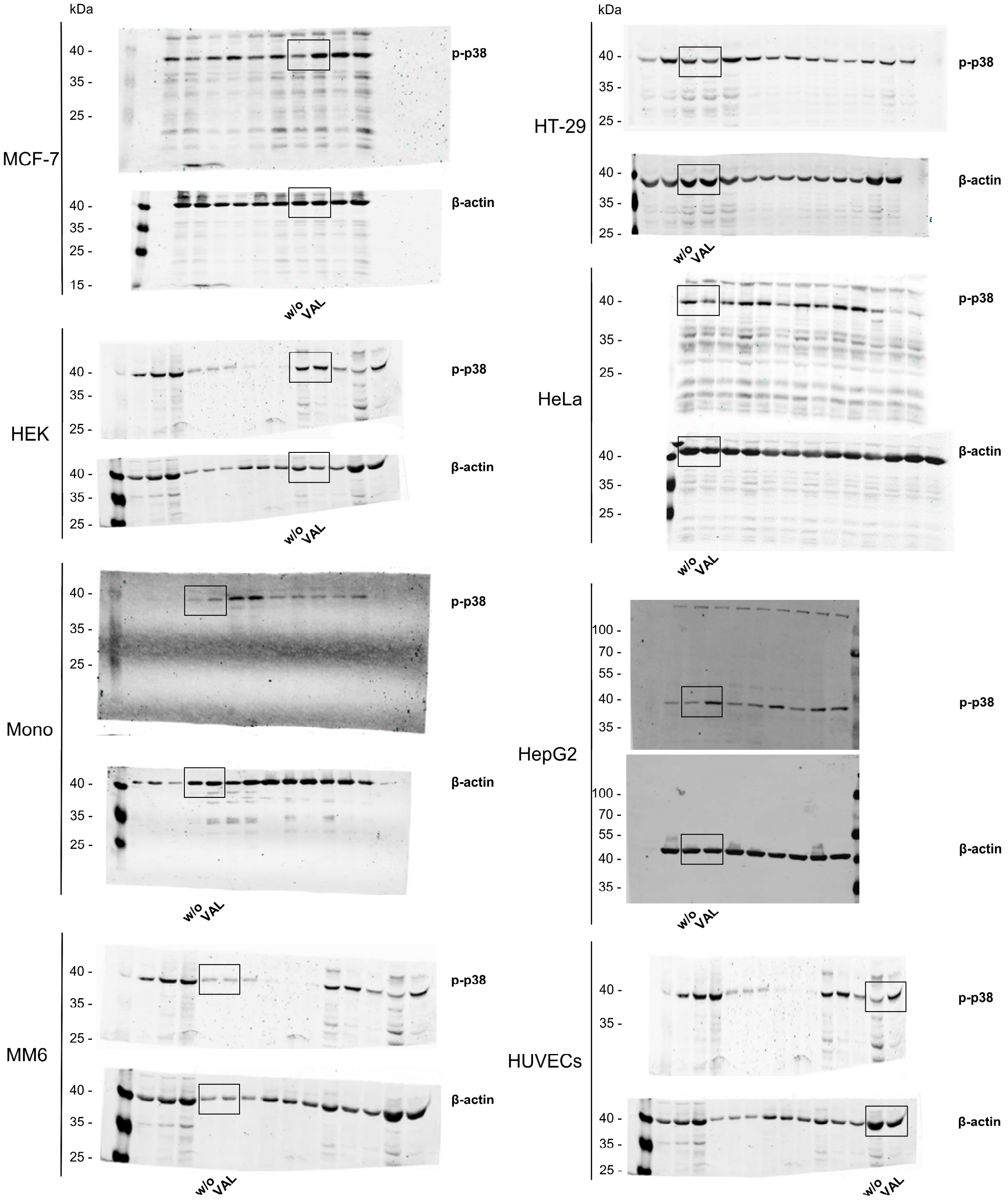

Supplementary Fig. 29 Uncropped versions of the blots presented in Supplementary Fig. 2b.

Fig. S4c

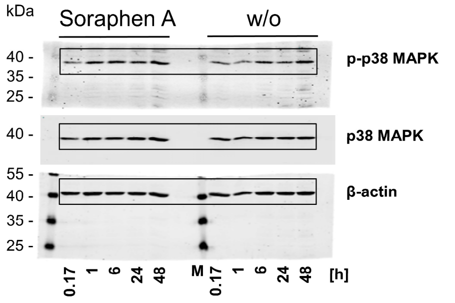

Fig. S4e

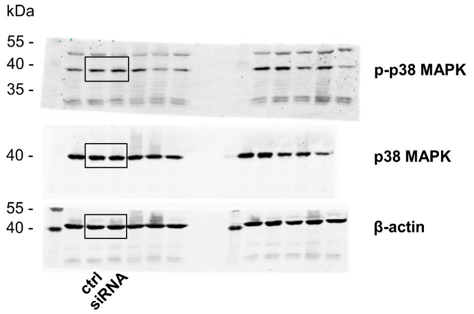

Fig. S9b

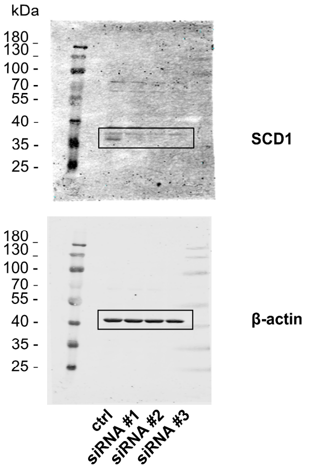

Supplementary Fig. 30 Uncropped versions of the blots presented in Supplementary Fig. 4c, 4e, 9b.

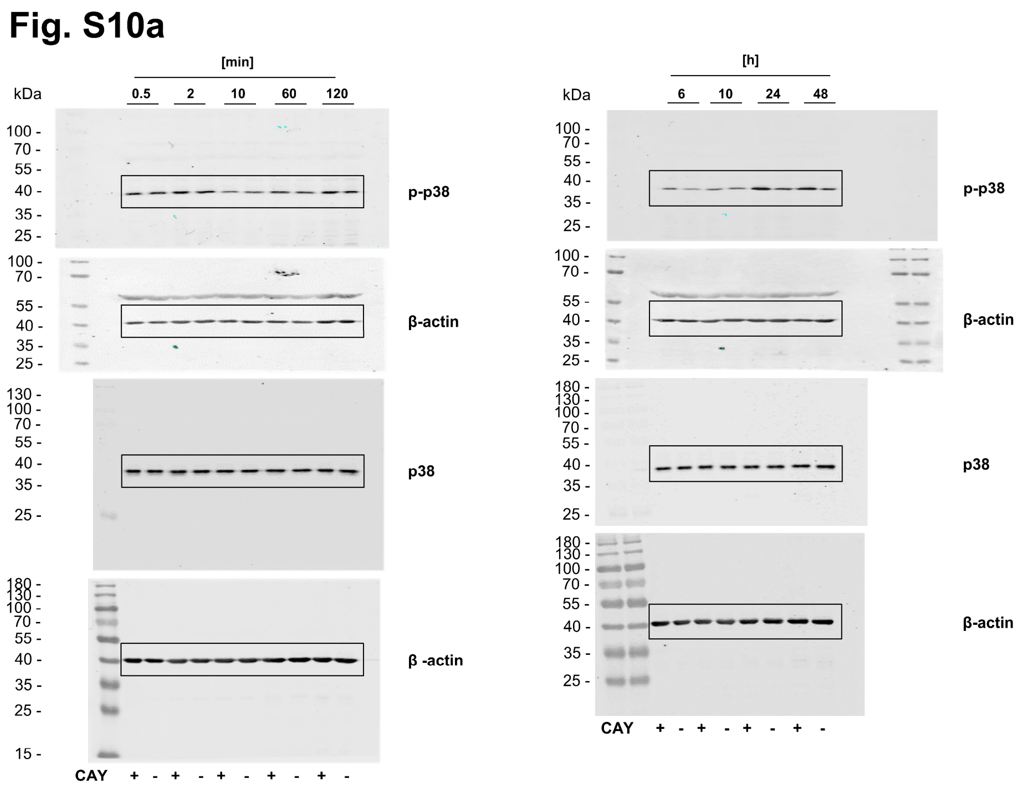

Supplementary Fig. 31 Uncropped versions of the blots presented in Supplementary Fig. 10a.

Fig. S10b

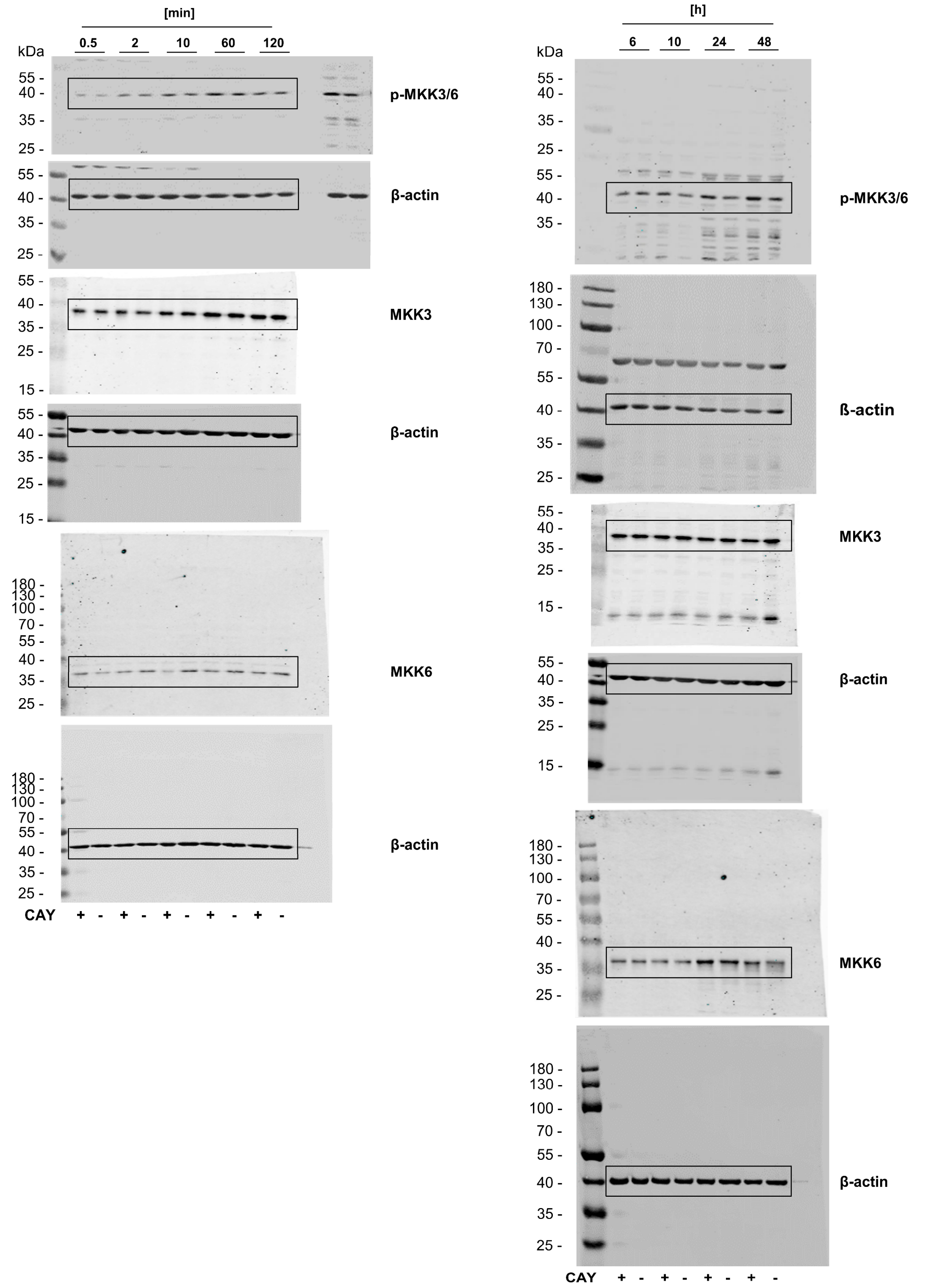

Supplementary Fig. 32 Uncropped versions of the blots presented in Supplementary Fig. 10b.

Fig. S10c

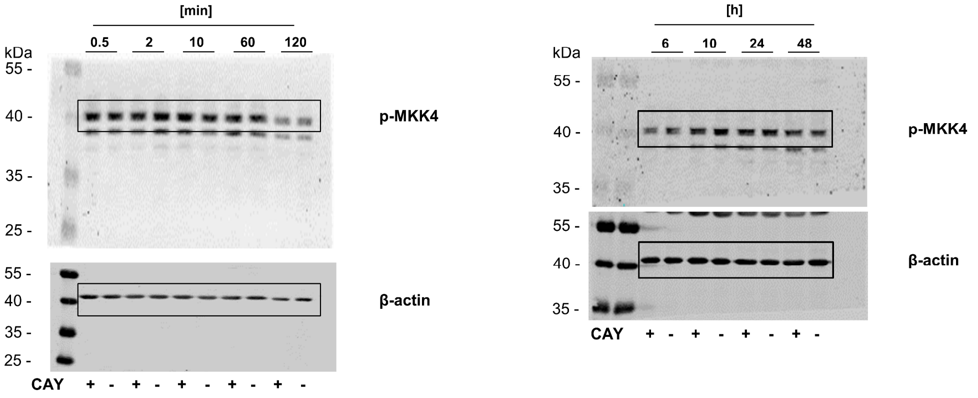

Fig. S10d

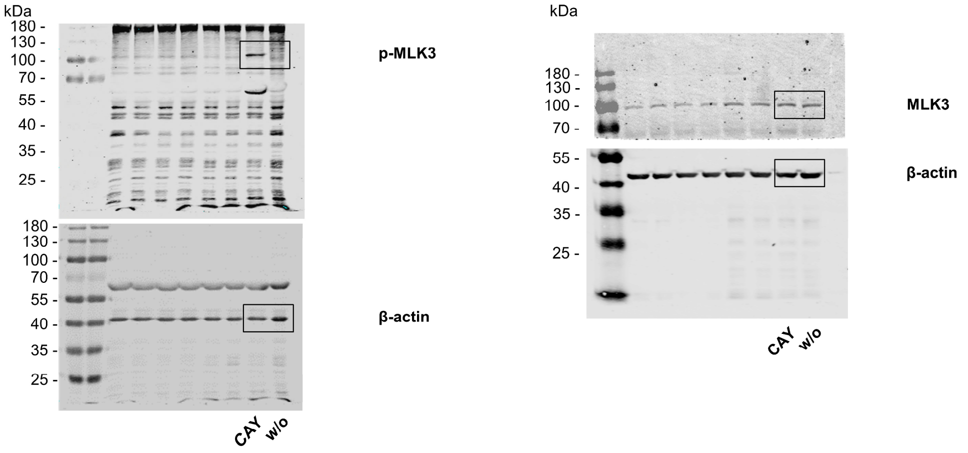

Supplementary Fig. 33 Uncropped versions of the blots presented in Supplementary Fig. 10c, 10d.

Fig. S14a

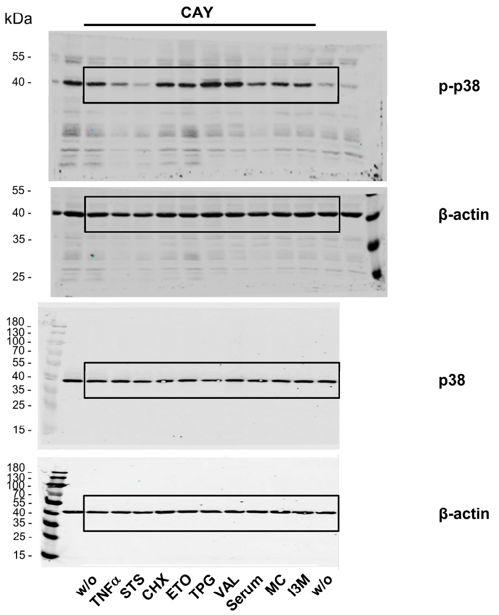

Fig. S14b

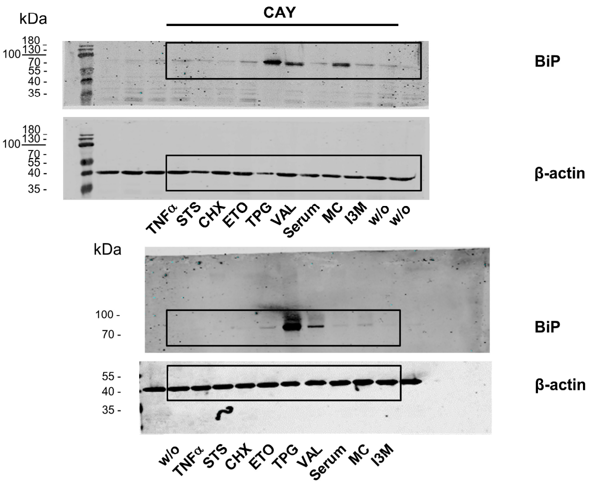

Fig. S14c

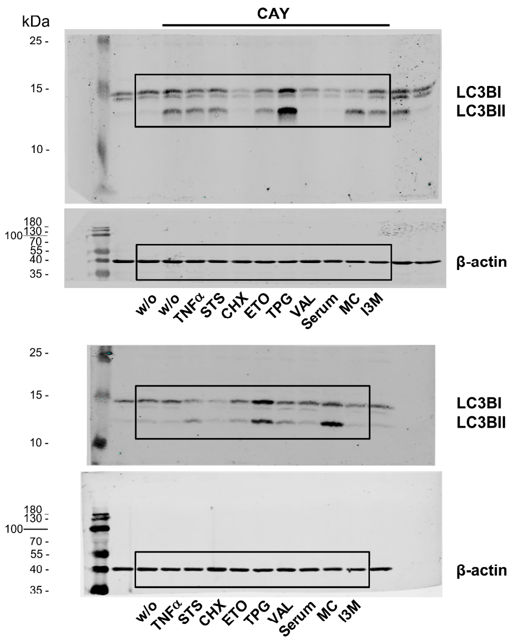

Fig. S14d

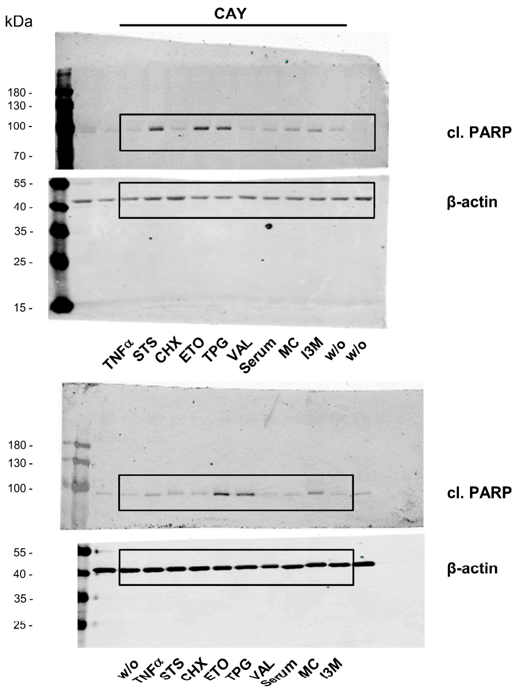

Supplementary Fig. 34 Uncropped versions of the blots presented in Supplementary Fig. 14a-d.

Fig. S16a

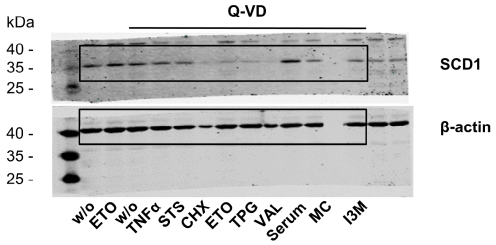

Fig. S16b

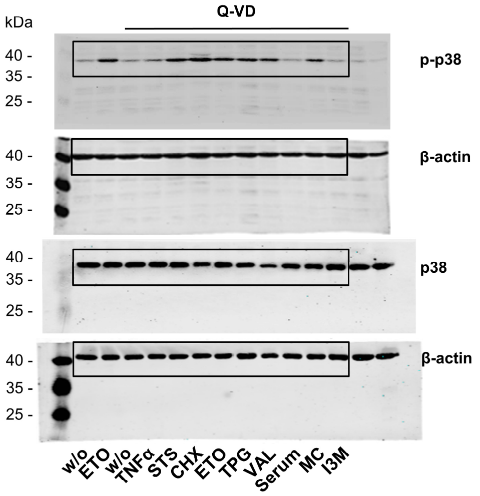

Fig. S17

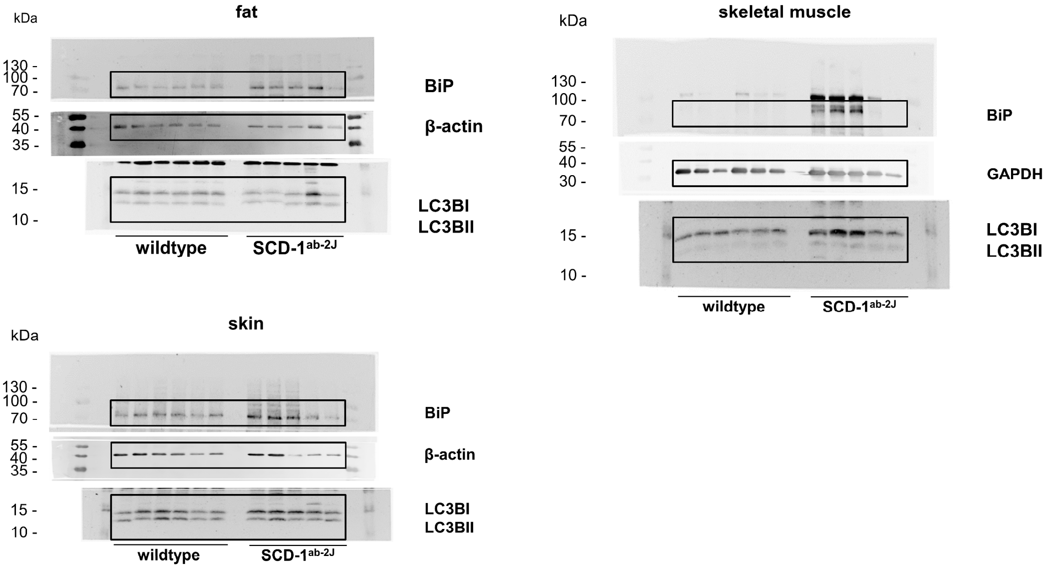

Supplementary Fig. 35 Uncropped versions of the blots presented in Supplementary Fig. 16a, 16b, 17.

Fig. S21b

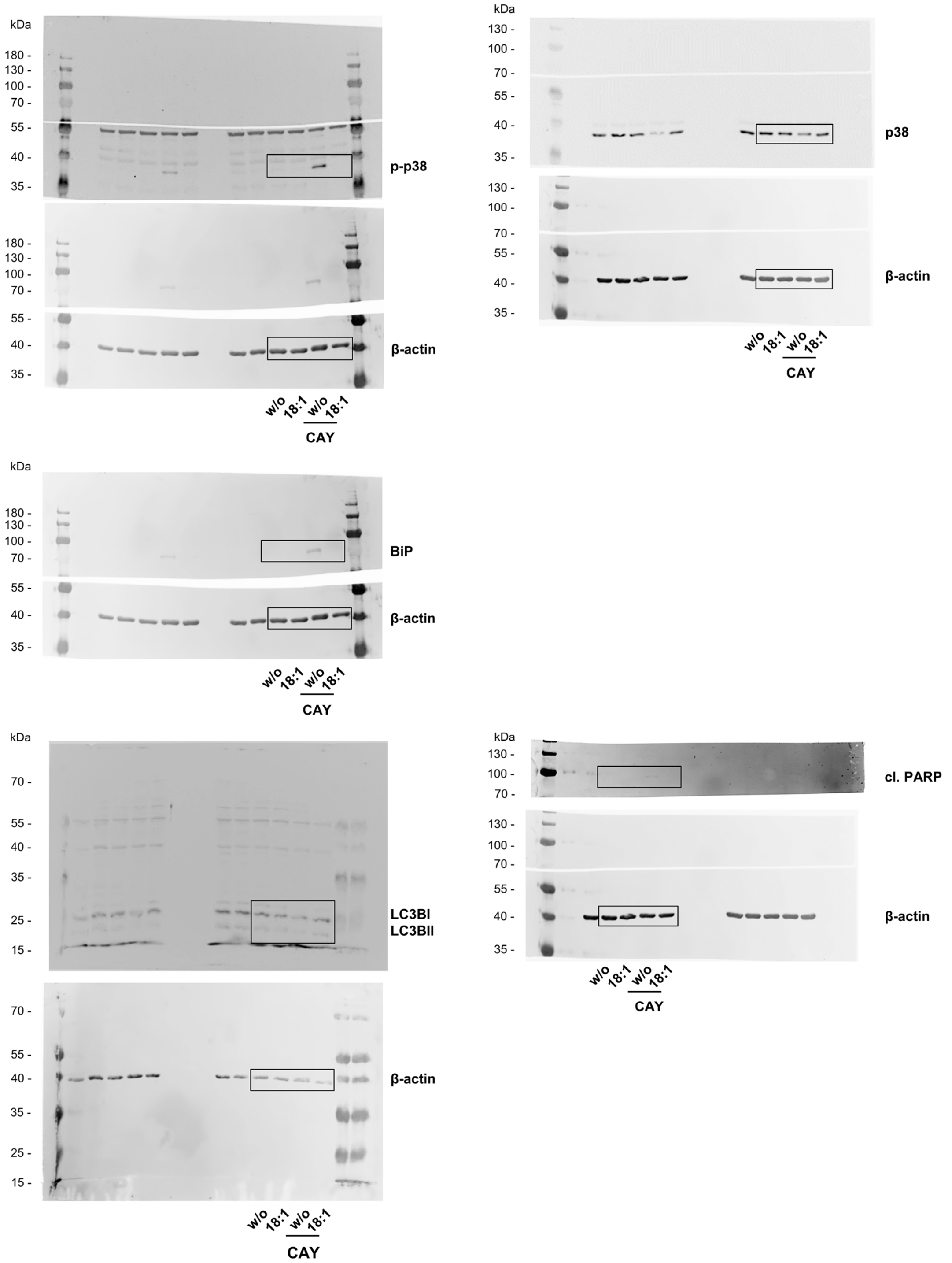

Supplementary Fig. 36 Uncropped versions of the blots presented in Supplementary Fig. 21b.

Fig. S26a

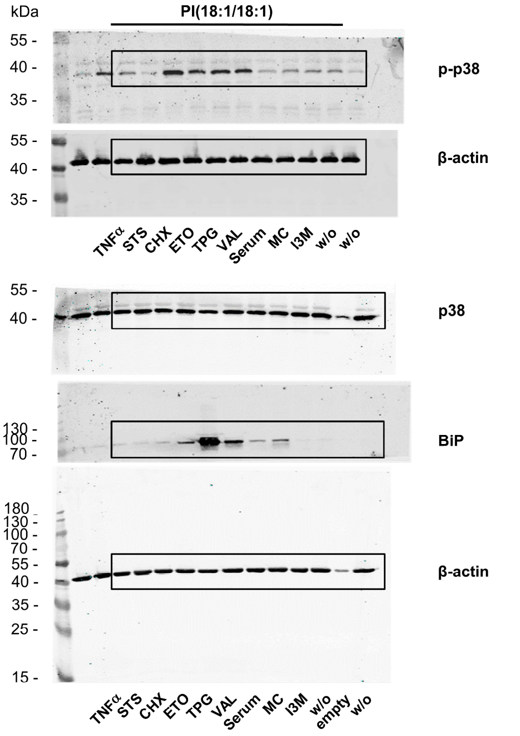

Fig. S26b

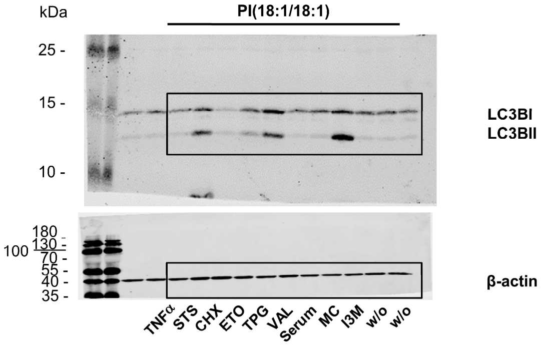

Fig. S26c

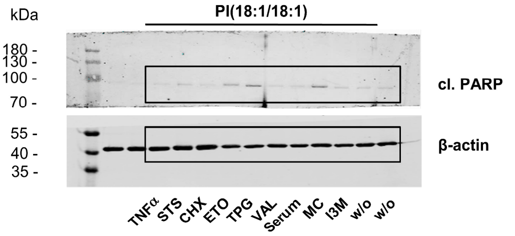

Supplementary Fig. 37 Uncropped versions of the blots presented in Supplementary Fig. 26a-c.

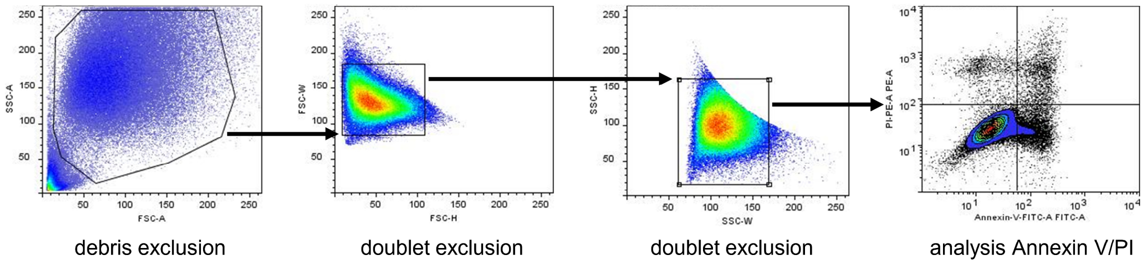

Supplementary Fig. 38 Gating strategy for Annexin V/propidium iodide staining.

Supplementary Tables

Supplementary Table 1 DEGs related to p38 MAPK activation and stress signaling in starved, *Smed-cct3A* silenced planarians

Category	Gene	***DEG*** [1] *(cct3A* RNAi *versus gfp* RNAi)	PlanMine ID [2]
Activators	*bcl3-1*	no	dd_Smed_v6_1555_0_1,<br>dd_Smed_v6_8253_0_1
	ecsit	no	dd_Smed_v6_5679_0_1,<br>dd_Smed_v6_5679_0_2
	hep	no	dd_Smed_v6_3580_0_1,<br>dd_Smed_v6_3580_0_2,<br>dd_Smed_v6_5234_0_1
	jnk	no	dd_Smed_v6_5924_0_1,<br>dd_Smed_v6_5924_0_2,<br>dd_Smed_v6_51510_0_1
	jun D	no	dd_Smed_v6_5875_0_1
	mkk4	no	dd_Smed_v6_3173_0_1
	mkk6-1	no	dd_Smed_v6_1286_0_1
	mkk6-2	no	dd_Smed_v6_2106_0_1
	p38-1	yes (upregulation)	dd_Smed_v6_1282
	p38-2	no	dd_Smed_v6_9252
	pgrp-1	yes (upregulation)	dd_Smed_v6_9550_0_1
	pgrp-2	no	d_Smed_v6_13624_0_1
	pgrp-3	no	dd_Smed_v6_6162_0_1
	pgrp-4	no	dd_Smed_v6_8505_0_1
	pgrp-5	no	dd_Smed_v6_1679_0_1
	pgrp-6	no	dd_Smed_v6_817_1_1
	pgrp-7	yes (upregulation)	dd_Smed_v6_1523_0_1
	tab1-1	no	dd_Smed_v6_3322_0_1
	tak1	no	dd_Smed_v6_2589_0_1,<br>dd_Smed_v6_2589_0_2,<br>dd_Smed_v6_2589_0_3
	traf2-1	no	dd_Smed_v6_3837_0_1
	traf2-2	no	dd_Smed_v6_4148_0_1
	traf5-1	no	dd_Smed_v6_19417_0_1
	traf6-1	no	dd_Smed_v6_11354_0_1
	traf6-3	no	dd_Smed_v6_2937_0_1
	ulk4	yes (upregulation)	dd_Smed_v6_7178_0_1
Suppressors	*cyld-1*	yes (downregulation)	dd_Smed_v6_3043_0_1
	pp6	no	dd_Smed_v6_581_0_1
	trabid	no	dd_Smed_v6_2288_0_1
	traf3-1	no	dd_Smed_v6_4392_0_1
	otud7b	no	dd_Smed_v6_10637_0_1
	xiap	yes (downregulation)	dd_Smed_v6_1803_0_2,<br>dd_Smed_v6_1803_0_1
Others	*bcl2-1*	no	dd_Smed_v6_1018_0_1
	bcl2-2	no	dd_Smed_v6_84197_0_1
	tehao	no	dd_Smed_v6_14454_0_1

[1] DEG, differentially regulated gene
[2] https://planmine.mpibpc.mpg.de/planmine/begin.do

Supplementary Table 2 Murine primer sequences used in quantitative RT-PCR experiments

Gene	Forward primer (5‘ → 3‘)	Reverse primer (5‘ → 3‘)
Scd1	CATCATTCTCATGGTCCTGCTG	AGCCGTGCCTTGTAAGTTCTGT
Actb	GCTGTGCTATGTTGCTCTAGACTT	AATTGAATGTAGTTTCATGGATGC
Gapdh	TGACAATGAATACGGCTACAGCA	CTCCTGTTATTATGGGGGTCTGG
Mapk14	TATGGCTCGGTGTGTGCTG	GTGACCTTGCGGGTGTGAAC
Hspa5	GTTCTTGCCATTCAAGGTGGTTG	AGCATCTTTGGTTGCTTGTCGC
Map1lc3b	ACAAAGAGTGGAAGATGTCCGGC	GCCCATTCACCAGGAGGAAGAAG
Parp1	AGCAGAAGGTCAAGAAGACGGC	ACATGCTCCTGTTGGACTTGGC
Cdipt	AGCATGAGCTTGGACGTGGC	GGTGAACAGAGCAGGCCTAGAG
Gapdh	CTCAGGAGAGTGTTTCCTCGTCC	CCGTTGAATTTGCCGTGAGTGG

Supplementary Notes

Supplementary Note 1

PI(18:1/18:1) ratio and p38 MAPK activation across cell lines

To study the association between MUFA-PI levels and p38 MAPK activation, we optimized the concentrations of cell death inducers in fibroblasts with the aim to induce initial morphological changes. The experimental settings were then transferred to other cell lines. Inverse correlations were observed for MCF-7, HEK-293, monocytes, and HepG2 cells (either reaching significance or by trend), whereas neither PI(18:1/18:1) levels nor p38 MAPK phosphorylation were affected in MM6, HeLa, and, given the variance, potentially also HT29 cells (**Fig. 2e**). While we cannot exclude that the functional link between PI(18:1/18:1) ratios and p38 MAPK activation is limited to distinct cell lines/types, we rather consider their different responsiveness to VAL being critical for the variance. Note that we applied the same VAL concentration independent of the sensitivity of the cell lines/types towards the cytotoxic drug. As expected from such an experimental design, i) a strong reduction of PI(18:1/18:1) was associated with a strong increase of p38 MAPK phosphorylation (MCF-7, HepG2), ii) a moderate reduction of PI(18:1/18:1) by trend correlated with a moderate increase of p38 MAPK activation again by trend (HEK293, monocytes), and ii) cell lines, whose PI(18:1/18:1) levels were non-responsive to VAL (at the applied concentration) did also not show p38 MAPK activation (MM6, HeLa, and potentially HT29). There is one exception, which we cannot readily explain, namely HUVEC, where we observed both a decrease of PI(18:1/18:1) ratios and p38 MAPK phosphorylation, though neither of the two effects reached significance. Together, our findings show a consistent correlation between PI(18:1/18:1) levels and p38 MAPK phosphorylation for most cell types/lines but do not answer the question whether the PI(18:1/18:1)-dependent regulation of

p38 MAPK is universal (for optimized concentrations and incubation times across cell lines) or limited to distinct cell lines/cell types.

Supplementary Note 2

Description of datasets shown in Fig. 2e

Mean + s.e.m. and single data from *n* = 2 (MM6 for p-p38 MAPK), *n* = 3 (HepG2, MCF-7, HUVEC, monocytes for PI; HT29, HEK293, MCF-7, monocytes for p-p38 MAPK), *n* = 4 (MM6 for PI; HepG2, HeLa, HUVEC for p-p38 MAPK), *n* = 7 (HEK293, HT29 for PI), *n* = 8 (HeLa for PI) independent experiments.

Supplementary Note 3

MUFA-PI depletion and stress signaling is independent of ACC suppression

Whether the apoptotic decrease of fatty acid biosynthesis is also responsible for the depletion of MUFA-PI was investigated using the selective ACC inhibitor soraphen A and by ACC silencing. Soraphen A lowered the total amount of phospholipids, as exemplarily shown for PC (**Supplementary Fig. 4a**), but did not affect MUFA-PC ratios within 48 h and even increased MUFA-PI ratios (**Supplementary Fig. 4b**), while phosphorylation of p38 MAPK was downregulated by trend (**Supplementary Fig. 4c**). Comparable results were obtained by ACC1 knockdown (**Supplementary Fig. 4d**, **e**). The knockdown efficiency of the *Acc1* siRNA has been reported before for NIH-3T3 fibroblasts (Pein, H. et al., in preparation).

Supplementary Note 4

Functional link between cytotoxic SCD1 depletion and stress signaling

We expected that the SCD1-dependent induction of cytotoxic phenotypes is already at the maximum in CAY10566-treated fibroblasts and that cytotoxic stimuli adding to these phenotypes will be less efficient in further enhancing the response, in case that the mechanism actually depends on SCD1. To visualize the difference in cellular responsiveness between cells with active and inactive SCD1, we normalized the effect of the cytotoxic conditions to vehicle and CAY10566-treated cells, respectively. In fact, our panel of cytotoxic inducers was less efficient to decrease MUFA-PI ratios (**Supplementary Fig. 13a**) and to enhance p38 MAPK activation (**Supplementary Fig. 13b**) in SCD1-inactivated cells. Moreover, CAY10566 diminished the capability of cytotoxic stimuli to further induce BiP expression (**Supplementary Fig. 13c**), LC3B cleavage and lipidation (**Supplementary Fig. 13d**), and PARP cleavage (**Supplementary Fig. 13e**), which suggests a central role of SCD1 in promoting ER stress, autophagy, and apoptotic progression.

Supplementary Note 5

p38 MAPK does not shape the phospholipid MUFA composition

To exclude that SCD1-dependent changes in the phospholipid composition are mediated by p38 MAPK, we treated fibroblasts with the selective p38 MAPK inhibitor Skepinone-L[2]. Neither MUFA-PI nor MUFA-PC ratios were markedly affected by the inhibitor (**Supplementary Fig. 15**) in line with our hypothesis that phospholipid-bound MUFAs act as SCD1-derived signaling lipids that suppress stress signaling.

Supplementary Note 6

Caspases are not essential for the early cytotoxic decrease of SCD1

Programmed cell death by apoptosis channels multifaceted cytotoxic mechanisms into the activation of specialized cysteine proteases (caspases), which trigger a controlled, non-inflammatory destruction of the cell[3]. Whether the diminished SCD1 expression early in programmed cell death depends on caspases, was investigated using the pan-caspase inhibitor (3*S*)-5-(2,6-difluorophenoxy)-3-[[(2*S*)-3-methyl-1-oxo-2-[(2-uinolinylcarbonyl)amino]butyl]-amino]-4-oxo-pentanoic acid (Q-VD-OPh)[4], which inhibits caspase 3 under our experimental conditions (Pein, H. et al., in preparation). Q-VD-OPh neither consistently prevented the cytotoxic drop in SCD1 (**Supplementary Fig. 16a**), the activation of p38 MAPK (**Supplementary Fig. 16b**) nor the depletion of phospholipid-bound MUFAs (**Supplementary Fig. 16c**) including PI(18:1/18:1) (**Supplementary Fig. 16d**). Thus, caspases are neither essential to decrease SCD1 expression nor to induce phospholipid remodeling and stress signaling during the onset of programmed cell death, even though they are exploited by specific cytotoxic settings. TPG and serum depletion, for example, engage a caspase-dependent mechanism to reduce MUFA-PI levels (**Supplementary Fig. 16c**, **d**). Moreover, p38 MAPK phosphorylation (**Supplementary Fig. 16b**) was diminished by Q-VD-OPh in serum-starved fibroblasts by trend.

Supplementary Note 7

Description of datasets shown in Fig. 6

Mean + s.e.m. (**e,** left panel) and single data (**a**, **b**, **d**, **e**, right panel, **g**, **h**) from n = 2 (**h** for control siRNA + PI(16:0/16:0)), n = 3 (**a**, right panel for PI(16:0/16:0) ± CAY, PI(18:0/20:4) ± CAY, **d**, **g**, right panel for PI(16:0/16:0) + CAY, PI(18:0/20:4) + CAY, **h** for control siRNA ± PI(18:1/18:1)), n =

4 (**a**, left panel, **b** for CAY, PI(16:0/16:0), **e**, **g**, left panel), *n* = 5 (**b** for w/o, PI(16:0/16:0) + CAY, PI(18:1/18:1) ± CAY), *n* = 6 (**a**, right panel for PC(16:0/16:0) ± CAY), *n* = 7 (**a**, right panel for PI(18:1/18:1) + CAY, **g**, right panel for w/o, CAY, PC(16:0/16:0) + CAY, PI(18:1/18:1) + CAY), *n* = 8 (**a**, right panel for w/o, CAY, PI(18:1/18:1)) independent experiments or *n* = 8 (**h** for siRNA + PI(16:0/16:0)), *n* = 9 (**h**) based on three different *Scd1* siRNA in three independent experiments.

Supplementary Note 8

Cytotoxic stress impacts the availability of enzymes in PI metabolism

Cell death induction by VAL and MC altered the cellular levels of phosphatidylinositol transfer proteins (Pitpn), which channel PI to diverse functional pools[5]. Pitpna was markedly up- and Pitpnb downregulated (**Supplementary Fig. 24a-d**). The SCD1 inhibitor CAY10566 selectively mimicked the effect on Pitpna (**Supplementary Fig. 24a**, **b**, **e**). The mechanism by which Pitpna is enriched seems not to rely on PI(18:1/18:1) depletion, as suggested from the failure of PI(18:1/18:1) supplementation to compensate for SCD1 inhibition (**Supplementary Fig. 24a**, **b**, **f**).

The consequences of cytotoxic stress on PI-derived PIPs are dominated by a drop in enzymes involved in PIP degradation, i.e., phosphatases (Sacm1l, Inppl1) and phospholipase Cβ3 (Plcb3) (**Supplementary Fig. 24a-d**). CAY10566 lowered the cellular levels of these proteins comparably to VAL and MC, and the decline was attenuated by PI(18:1/18:1) for phosphatidylinositide phosphatase SAC1 (Sacm1l) and phosphatidylinositol-3,4,5-trisphosphate 5-phosphatase 2 (Inppl1) (**Supplementary Fig. 24a**, **b**, **e**, **f**).

Supplementary Note 9

Regulation of the stress proteome by SCD1/PI(18:1/18:1)

p38 MAPK signaling

Studies on signal-induced proliferation-associated protein (Sipa)1-deficient cells[6] suggest that the drop in Sipa1 (**Fig. 8a**), which activates the GTPase activity of Rap1 and Rap2[7], might add to cytotoxic p38 MAPK activation. Cellular levels of Sipa1 were reduced by cytotoxic stress and SCD1 inhibition, and PI(18:1/18:1) and slightly less PI(16:0/16:0) compensated for the loss of SCD1 activity (**Fig. 8a**). As expected from an activation of p38 MAPK, the expression of the target gene latexin (Lxn), which is repressed by p38 MAPK, followed an inverse pattern (**Fig. 8a**)[8]. Counter-regulatory mechanisms seem to be initiated to buffer p38 MAPK activation, e.g., by repressing i) the serine/threonine-protein kinase TAO1 (Taok1), ii) MKK3 (Map2k3)[9], iii) 5'-AMP-activated protein kinase (AMPK, Prkaa1), and iv) Cdc42 (**Fig. 8a**). Taok1 is a MAP3K that initiates the phosphorylation cascade towards p38 MAPK[9], AMPK recruits p38 MAPK to the scaffold protein Tab1 for autophosphorylation[10], and Cdc42 is a small GTPase of the Rho family that, among its pleiotropic activities, contributes to the activation of p38 MAPK[11].

Unfolded protein response

By dephosphorylating the ER transmembrane kinase/endoribonuclease Ire1α, Ppp2ca deactivates the intrinsic RNase activity of the ER sensor, thereby preventing Xbp1 mRNA processing[12]. The reduced cellular availability of Ppp2ca upon cytotoxic stress and SCD1 inhibition (**Fig. 8a-c**) is thus expected to activate the Ire1α/Xbp1 branch of the UPR. The UPR markers BiP (Hspa5) and ERO1-like protein alpha (Ero1a) are consequently upregulated (**Fig. 8a-c**).

Programmed cell death

Cytotoxic caspase activation seems to be facilitated by the depletion of baculoviral IAP repeat-containing protein 6 (Apollo/Birc6), a member of the inhibitor of apoptosis (IAP) family that suppresses caspases 8 and 9 activation[13]. The levels of major caspase substrates (Bid, plectin, Bcap31, Parp3) accordingly decreased, and PI(18:1/18:1) attenuated this decline (**Fig. 8a**, **b**). Interestingly, Bcap31 is also an important sensor for ER-mitochondria communication during ER stress[14], which points towards another SCD1/PI(18:1/18:1)-regulated crosslink between the here investigated stress(-adaptive) pathways.

Supplementary Note 10

Smed-cct3A knockdown in starved planarians

We analyzed the transcriptome of starved planarians to investigate the effect of *Smed-cct3A* silencing on p38 MAPK activation and apoptosis induction. *cct3A* RNAi i) elevated p38-1 expression (a planarian homologue of p38 MAPK) and ii) upregulated peptidoglycan recognition receptors (**Fig. 9h**), which activate or sensitize cells towards stress signaling (among others via p38 MAPK)[15,16] but are also induced by p38 MAPK as part of a feed-back mechanism[17]. Moreover, *cct3A* knockdown iii) slightly decreased the mRNA levels of ubiquitin carboxy-terminal hydrolase (*cyld-1*) (**Fig. 9h**), which deubiquitinates Tak1, inhibits the activation of Jnk-p38 MAPK cascades[18], and suppresses nuclear factor (NF)κB signaling[19,20]. iv) p38 MAPK activation seems to be further enhanced by the repression of *ulk4* (**Fig. 9h**), which limits the phosphorylation of stress-activated protein kinases (including p38 MAPK)[21]. Regarding possible links to apoptosis, we v) found X-linked inhibitor of apoptosis protein (*xiap*) being downregulated (**Fig. 9h**).

Supplementary Note 11

Diversity of mechanisms lowering the cellular PI(18:1/18:1) ratio

The mechanisms by which cytotoxic agents decrease PI(18:1/18:1) ratios in fibroblasts are diverse. For example, STS and I3M efficiently lower PI(18:1/18:1) ratios without substantially affecting SCD1 expression. Other SCD isoenzymes contribute as well to the biosynthesis of MUFAs, which are incorporated into PI by lysophosphatidylinositol acyltransferases and released by a set of phospholipases[22,23]. While we have previously shown that SCD2 is highly expressed in NIH-3T3 fibroblasts[1], established lysophosphatidylinositol acyltransferases lack selectivity for 18:1[23] and seem therefore not be capable to selectively manipulate PI(18:1/18:1) ratios. Distinct phospholipases such as phospholipases A_2 and C[23,24], on the other hand, preferentially release PUFAs from phospholipids, which might indirectly favor MUFA enrichment. In particular phospholipase C in concert with diacylglycerol lipase and monoacylglycerol lipase forms an efficient system for the release of PUFAs from PI[24].

Supplementary Note 12

Kinetics of PI(18:1/18:1) depletion and p38 MAPK activation

The decline in PI(18:1/18:1) (**Fig. 1c**) upon cytotoxic stress largely correlates with the second phase of p38 MAPK activation from 6 to 48 h (**Fig. 2b**). In the following, we briefly discuss apparent exceptions:

p38 MAPK phosphorylation following treatment with VAL significantly increased up to 6 h (3.4-fold increase, $P = 0.04$; phase I) and then decreased again at 24 h by trend (2.8-fold

increase) before strongly and significantly rising again at 48 h (4.1-fold increase, phase II). The second phase of p38 MAPK activation correlates with a decrease in PI(18:1/18:1) levels.

ETO decreased PI(18:1/18:1) levels (17%, $P > 0.99$) and increased p38 MAPK phosphorylation (3.3-fold, $p = 0.09$) at 24 h. While the depletion of PI(18:1/18:1) is less pronounced as we would expect from p38 MAPK phosphorylation, interpretations should be made with caution considering the non-significant effects at this time point. Interestingly, ETO is the only cytotoxic stressor that did not evoke p38 MAPK activation within 6 h, and it is tempting to speculate that ETO shows a delayed phase I response that might overlap at 24 h with the phase II activation of p38 MAPK.

Serum depletion decreased PI(18:1/18:1) levels by 78% and increased p38 MAPK phosphorylation 2.1-fold at 48 h, which is relatively low when compared to other cytotoxic stressors. Considering that p38 MAPK activation is an ATP-dependent process, one possible explanation for the poor activation of p38 MAPK might be the low energy/ATP status of fibroblasts upon serum depletion, as indicated by the substantial increase of the ADP/ATP ratio (Pein, H. et al., in preparation).

STS decreased PI(18:1/18:1) levels and strongly induced p38 MAPK phosphorylation (see source data for Fig. 2b). However, we decided to grey out these results in **Fig. 2b** because the compound is a pan-kinase inhibitor and hyperphosphorylation of p38 MAPK might therefore not necessarily indicate an activation.

Supplementary Note 13

MUFA-PI candidates with potential stress-reducing activity

Whether other MUFA-PIs share the lipokine activity of PI(18:1/18:1) is still elusive because the respective phospholipids are not commercially available. PI(16:1/18:1) contains, like PI(18:1/18:1), two MUFAs and is comparably regulated by SCD1 in programmed cell death, which renders PI(16:1/18:1) as likely candidate to share the bioactive character. The incorporation of 16:1 into a lipokine-like molecule is of particular interest because 16:1 has been described as bioactive signaling factor that links adipocytes with systemic metabolism[25] and induces proliferation of fibroblasts[26]. The hypothesis that 16:1, at least partially, mediates its hormonal activity through PI-bound 16:1 is supported by a recent study that describes an efficient cellular uptake and subsequent incorporation of 16:1 into PI, yielding PI(16:0/16:1) and PI(16:1/18:1) as major species[26]. Future studies are needed to fully explore the physiological relevance and diagnostic value of PI(18:1/18:1) and PI(16:1/18:1) in SCD1-related diseases.

Supplementary References

1 Koeberle, A. *et al.* Role of p38 mitogen-activated protein kinase in linking stearoyl-CoA desaturase-1 activity with endoplasmic reticulum homeostasis. *FASEB J* **29**, 2439-2449 (2015).

2 Koeberle, S. C. *et al.* Skepinone-L is a selective p38 mitogen-activated protein kinase inhibitor. *Nat Chem Biol* **8**, 141-143 (2012).

3 Riedl, S. J. & Shi, Y. Molecular mechanisms of caspase regulation during apoptosis. *Nat Rev Mol Cell Biol* **5**, 897-907 (2004).

4 Caserta, T. M., Smith, A. N., Gultice, A. D., Reedy, M. A. & Brown, T. L. Q-VD-OPh, a broad spectrum caspase inhibitor with potent antiapoptotic properties. *Apoptosis* **8**, 345-352 (2003).

5 Grabon, A., Bankaitis, V. A. & McDermott, M. I. The interface between phosphatidylinositol transfer protein function and phosphoinositide signaling in higher eukaryotes. *J Lipid Res* **60**, 242-268 (2019).

6 Ishida, D. *et al.* Rap1 signal controls B cell receptor repertoire and generation of self-reactive B1a cells. *Immunity* **24**, 417-427 (2006).

7 Kurachi, H. *et al.* Human SPA-1 gene product selectively expressed in lymphoid tissues is a specific GTPase-activating protein for Rap1 and Rap2. Segregate expression profiles from a rap1GAP gene product. *J Biol Chem* **272**, 28081-28088 (1997).

8 Zer, C., Sachs, G. & Shin, J. M. Identification of genomic targets downstream of p38 mitogen-activated protein kinase pathway mediating tumor necrosis factor-alpha signaling. *Physiol Genomics* **31**, 343-351 (2007).

9 Wagner, E. F. & Nebreda, A. R. Signal integration by JNK and p38 MAPK pathways in cancer development. *Nat Rev Cancer* **9**, 537-549 (2009).

10 Lanna, A., Henson, S. M., Escors, D. & Akbar, A. N. The kinase p38 activated by the metabolic regulator AMPK and scaffold TAB1 drives the senescence of human T cells. *Nature immunology* **15**, 965-972 (2014).

11 Maldonado, M. D. M. & Dharmawardhane, S. Targeting Rac and Cdc42 GTPases in Cancer. *Cancer Res* **78**, 3101-3111 (2018).

12 Hetz, C., Zhang, K. & Kaufman, R. J. Mechanisms, regulation and functions of the unfolded protein response. *Nat Rev Mol Cell Biol* **21**, 421-438 (2020).

13 Hao, Y. *et al.* Apollon ubiquitinates SMAC and caspase-9, and has an essential cytoprotection function. *Nat Cell Biol* **6**, 849-860 (2004).

14 Namba, T. BAP31 regulates mitochondrial function via interaction with Tom40 within ER-mitochondria contact sites. *Sci Adv* **5**, eaaw1386 (2019).

15 Torre, C. *et al.* Staphylococcus aureus Promotes Smed-PGRP-2/Smed-setd8-1 Methyltransferase Signalling in Planarian Neoblasts to Sensitize Anti-bacterial Gene Responses During Re-infection. *EBioMedicine* **20**, 150-160 (2017).

16 Ha, E. M. *et al.* Coordination of multiple dual oxidase-regulatory pathways in responses to commensal and infectious microbes in drosophila gut. *Nature immunology* **10**, 949-957 (2009).

17 Wang, H., Gupta, D., Li, X. & Dziarski, R. Peptidoglycan recognition protein 2 (N-acetylmuramoyl-L-Ala amidase) is induced in keratinocytes by bacteria through the p38 kinase pathway. *Infect. Immun.* **73**, 7216-7225 (2005).

18 Ji, Y. X. *et al.* The deubiquitinating enzyme cylindromatosis mitigates nonalcoholic steatohepatitis. *Nat Med* **24**, 213-223 (2018).

19 Trompouki, E. *et al.* CYLD is a deubiquitinating enzyme that negatively regulates NF-kappaB activation by TNFR family members. *Nature* **424**, 793-796 (2003).

20 Kovalenko, A. *et al.* The tumour suppressor CYLD negatively regulates NF-kappaB signalling by deubiquitination. *Nature* **424**, 801-805 (2003).

21 Lang, B. *et al.* Recurrent deletions of ULK4 in schizophrenia: a gene crucial for neuritogenesis and neuronal motility. *J Cell Sci* **127**, 630-640 (2014).

22 Hodson, L. & Fielding, B. A. Stearoyl-CoA desaturase: rogue or innocent bystander? *Prog Lipid Res* **52**, 15-42 (2013).

23 Hishikawa, D., Hashidate, T., Shimizu, T. & Shindou, H. Diversity and function of membrane glycerophospholipids generated by the remodeling pathway in mammalian cells. *J Lipid Res* **55**, 799-807 (2014).

24 Majerus, P. W. Arachidonate metabolism in vascular disorders. *J Clin Invest* **72**, 1521-1525 (1983).

25 Cao, H. *et al.* Identification of a lipokine, a lipid hormone linking adipose tissue to systemic metabolism. *Cell* **134**, 933-944 (2008).

26 Koeberle, A., Shindou, H., Harayama, T. & Shimizu, T. Palmitoleate is a mitogen, formed upon stimulation with growth factors, and converted to palmitoleoyl-phosphatidylinositol. *J Biol Chem* **287**, 27244-27254 (2012).
