# Supplementary material for: PI(18:1/18:1) is a SCD1-derived lipokine that limits stress signaling
Source: Nat Commun. 2022 May 27;13:2982. doi: 10.1038/s41467-022-30374-9 (PMC9142606; doi:10.1038/s41467-022-30374-9)
Supplement: Supplementary file 3 — Description of Additional Supplementary Files [file 41467_2022_30374_MOESM3_ESM.pdf]

## Description of Additional Supplementary Files

# PI(18:1/18:1) is a SCD1-derived lipokine that limits stress signaling

Thuermer et al.

**Supplementary Files**

**Supplementary Data 1** │ Processed proteomics data

Exported and processed data (stress proteomics, target fishing).

**Supplementary Data 2** │ Proteomic analysis of PI biosynthesis and metabolism

List of proteins considered for the analysis of PI biosynthesis and metabolism and of those qualifying for Supplementary Fig. 24.

**Supplementary Data 3** │ Proteomic analysis of p38 MAPK activation and stress signaling

List of proteins considered for the analysis of p38 MAPK activation and stress signaling.

**Supplementary Data 4** │ Proteomic analysis of ER stress, UPR and ERAD

List of proteins considered for the analysis of ER stress, UPR and ERAD.

**Supplementary Data 5** │ Proteomic analysis of autophagy pathways

List of proteins considered for the analysis of autophagy pathways.

**Supplementary Data 6** │ Proteomic analysis of apoptotic pathways

List of proteins considered for the analysis of apoptotic pathways.
