# Supplementary material for: PI(18:1/18:1) is a SCD1-derived lipokine that limits stress signaling
Source: Nat Commun. 2022 May 27;13:2982. doi: 10.1038/s41467-022-30374-9 (PMC9142606; doi:10.1038/s41467-022-30374-9)
Supplement: Supplementary file 10 — Reporting Summary [file 41467_2022_30374_MOESM10_ESM.pdf]

nature research

Corresponding author(s): Andreas Koeberle

Last updated by author(s): Mar 30, 2022

Reporting Summary

Nature Research wishes to improve the reproducibility of the work that we publish. This form provides structure for consistency and transparency in reporting. For further information on Nature Research policies, see our Editorial Policies and the Editorial Policy Checklist.

Statistics

For all statistical analyses, confirm that the following items are present in the figure legend, table legend, main text, or Methods section.

n/a	Confirmed	
☐	☒	The exact sample size (*n*) for each experimental group/condition, given as a discrete number and unit of measurement
☐	☒	A statement on whether measurements were taken from distinct samples or whether the same sample was measured repeatedly
☐	☒	The statistical test(s) used AND whether they are one- or two-sided<br>*Only common tests should be described solely by name; describe more complex techniques in the Methods section.*
☒	☐	A description of all covariates tested
☐	☒	A description of any assumptions or corrections, such as tests of normality and adjustment for multiple comparisons
☐	☒	A full description of the statistical parameters including central tendency (e.g. means) or other basic estimates (e.g. regression coefficient) AND variation (e.g. standard deviation) or associated estimates of uncertainty (e.g. confidence intervals)
☐	☒	For null hypothesis testing, the test statistic (e.g. *F*, *t*, *r*) with confidence intervals, effect sizes, degrees of freedom and *P* value noted<br>*Give P values as exact values whenever suitable.*
☒	☐	For Bayesian analysis, information on the choice of priors and Markov chain Monte Carlo settings
☒	☐	For hierarchical and complex designs, identification of the appropriate level for tests and full reporting of outcomes
☐	☒	Estimates of effect sizes (e.g. Cohen's *d*, Pearson's *r*), indicating how they were calculated

Our web collection on statistics for biologists contains articles on many of the points above.

Software and code

Policy information about availability of computer code

Data collection	Analyst 1.6 and 1.7 (Sciex), AxioVision 4.8 (Carl Zeiss), BD FACSDiva 8.0.1 (BD Biosciences), Evolution-Capt Edge Software Version 18.06 (Vilber Lourmat), Laser Scanning Microscope LSM 510 Release Version 4.2 SP1 (Carl Zeiss), MassLynx v4.1 software (Waters), Mx3005P v4.10 software (Agilent Technologies), Odyssey Infrared Imaging System Application Software Version 3.0, Orbitrap Tribrid Series Tune software 3.5 (Thermo Fisher Scientific), qPCRsoft v4.1.3.0 software (Analytik Jena), SkanIt-Software (Version 2.4.4.5, Thermo Fisher Scientific), SoftMax Pro 7.1 (Molecular Devices), Vi-Cell XR Cell Viability Analyzer 2.03 and 2.06.3 (Beckman Coulter)
Data analysis	Analyst 1.6 and Analyst 1.7 (Sciex), Bio-1D Version 15.08c (Vilber Lourmat), BD FACSDiva 8.0.1 (BD Biosciences), Cytoscape 3.3 (Cytoscape Consortium), FlowJo v10 software (BD Biosciences), FlowLogic software V7 (Miltenyi Biotech), GraphPad InStat 3.10 (GraphPad Software), GraphPad Prism 8.0 and GraphPad Prism 9.0 (GraphPad Software), Microsoft Office Professional Plus 2016 (Microsoft), Morpheus (https://software.broadinstitute.org/morpheus, based on recent source code found at https://github.com/cmap/morpheus.js), Odyssey Infrared Imaging System Application Software Version 3.0, Origin 2020 (OriginLab), ProteinLynx Global Server (PLGS) version 2.5.2 (Waters) with Ion accounting Algorithm according to Li, G. Z. et al. Database searching and accounting of multiplexed precursor and product ion spectra from the data independent analysis of simple and complex peptide mixtures. Proteomics 9, 1696-1719 (2009), ProteomeDiscoverer 2.4 (Thermo Fisher Scientific), qPCRsoft v4.1.3.0 software (Analytik Jena), RStudio version 1.4.1106 (https://www.rstudio.com/) with the packages rstatix (version 0.7.0, https://cran.r-project.org/web/packages/rstatix/index.html) and ComplexHeatmap (version 3.14, https://www.bioconductor.org/packages/release/bioc/html/ComplexHeatmap.html), SigmaPlot 13 and SigmaPlot 14 (Systat Software GmbH).

For manuscripts utilizing custom algorithms or software that are central to the research but not yet described in published literature, software must be made available to editors and reviewers. We strongly encourage code deposition in a community repository (e.g. GitHub). See the Nature Research guidelines for submitting code & software for further information.

Data

Policy information about availability of data

All manuscripts must include a data availability statement. This statement should provide the following information, where applicable:

- Accession codes, unique identifiers, or web links for publicly available datasets
- A list of figures that have associated raw data
- A description of any restrictions on data availability

The mass spectrometry lipidomics data generated in this study have been deposited in the Metabolomics Workbench database (an international repository for metabolomics data and metadata, metabolite standards, protocols, tutorials and training, and analysis tools; Sud, M. et al. Metabolomics Workbench: An international repository for metabolomics data and metadata, metabolite standards, protocols, tutorials and training, and analysis tools. Nucleic Acids Res. 44, D463-470 (2016)) under accession code ST001740 [http://dx.doi.org/10.21228/M8Q39V]. The mass spectrometry proteomics data have been deposited to the ProteomeXchange Consortium via the PRIDE (Perez-Riverol, Y. et al. PRIDE Inspector Toolsuite: Moving Toward a Universal Visualization Tool for Proteomics Data Standard Formats and Quality Assessment of ProteomeXchange Datasets. Mol Cell Proteomics 15, 305-317 (2016)) partner repository with the dataset identifier PXD025396 [http://www.ebi.ac.uk/pride/archive/projects/PXD025396] and PXD031890 [http://www.ebi.ac.uk/pride/archive/projects/PXD031890]. All other data generated or analyzed during this study are provided in this published article, the Source Data, Supplementary Information, or Supplementary Data.

Field-specific reporting

Please select the one below that is the best fit for your research. If you are not sure, read the appropriate sections before making your selection.

- [x] Life sciences
- [ ] Behavioural & social sciences
- [ ] Ecological, evolutionary & environmental sciences

For a reference copy of the document with all sections, see nature.com/documents/nr-reporting-summary-flat.pdf

Life sciences study design

All studies must disclose on these points even when the disclosure is negative.

Sample size	Sample size was not pre-determined by statistical methods but was estimated based on previous studies. Cell-based studies on the lipid profile, stress-signaling, ER-stress/UPR and cell death were at least performed in three independent biological experiments, as described before (Koeberle, A. et al. Role of p38 mitogen-activated protein kinase in linking stearoyl-CoA desaturase-1 activity with endoplasmic reticulum homeostasis. FASEB J 29, 2439-2449 (2015) and Bartel, K. et al. Connecting lysosomes and mitochondria - a novel role for lipid metabolism in cancer cell death. Cell Commun Signal 17, 87 (2019)). Investigations related to the lipid profile and biomarkers in mice homozygous for the Scd1ab-2J allele were based on tissues from at least five animals. The sample size was estimated based on previous experiments (Pein, H. et al. Vitamin A regulates Akt signaling through the phospholipid fatty acid composition. FASEB J 31, 4566-4577 (2017)). For experiments that evaluate the role of PI(18:1/18:1) in systems with varying stress tolerance (e.g., IgM- and IgM+ B-cells and B-cell lymphoma from Eμ-Myc-transgenic mice, planaria with impaired ER stress adaption), sample sizes were selected based on following studies: Koeberle, A. et al. Role of p38 mitogen-activated protein kinase in linking stearoyl-CoA desaturase-1 activity with endoplasmic reticulum homeostasis. FASEB J 29, 2439-2449 (2015); Bartel, K. et al. Connecting lysosomes and mitochondria - a novel role for lipid metabolism in cancer cell death. Cell Commun Signal 17, 87 (2019); Pein, H. et al. Vitamin A regulates Akt signaling through the phospholipid fatty acid composition. FASEB J 31, 4566-4577 (2017); Gutierrez-Gutierrez, O. et al. Regeneration in starved planarians depends on TRiC/CCT subunits modulating the unfolded protein response. EMBO Rep 22, e52905 (2021); Joshi, G. et al. Dichotomous Impact of Myc on rRNA Gene Activation and Silencing in B Cell Lymphomagenesis. Cancers (Basel) 12 (2020). The specific number of independent biological experiments is given in the corresponding figure legend.
Data exclusions	Significant outliers ($P < 0.05$) were excluded from the analyses in Figs. 2c, 2d, 3e, 6a, 6h and Supplementary Figs. 13b, 16a, 16b and 20c using a Grubb's test (significance level of alpha = 0.05) but not from other figures. Unequal numbers of data sets in these analyses derive from different designs, samples not complying to our quality criteria, or loss of sample.
Replication	The replication of the data was successful. Data were generated in independent experiments or are representative for multiple animals.<br><br>1) Independent experiments on cultured or isolated cells: n ≥ 3 (except Fig. 7b: n = 1)<br>2) Analyses of mouse tissues (liver, fat, muscle, skin, brain, (pre)tumors, tibia) and serum are based on n = 5 (Scd1ab-2J), n = 6 (wildtype, periodontal infection for tibia/serum), or n = 4-6 (B-cell lymphoma) mice (one experiment).<br>3) Independent experiments on planaria (Schmidtea mediterranea): n = 3 (five planarians per experiment)<br><br>Detailed information about the exact number of experiments is given in the figure legends.
Randomization	Flasks/wells with seeded cells or animals were randomly assigned to treatment groups. Quantitative proteomics sample were run in a randomized order.
Blinding	Samples were not blinded. Rational: a) Readouts were quantitatively assessed. b) Sample preparation directly followed individual cell treatment by the same operator and was subjected to strict time schedules, which was poorly compatible to blinding. c) Experiments have been conducted following established protocols leaving no room for biased analysis. d) The data collection and technical analysis of cell counts, cell viability, lipids, and proteoms has been automatically conducted and manually controlled, which further minimized biased analysis.

Reporting for specific materials, systems and methods

We require information from authors about some types of materials, experimental systems and methods used in many studies. Here, indicate whether each material, system or method listed is relevant to your study. If you are not sure if a list item applies to your research, read the appropriate section before selecting a response.

Materials & experimental systems

n/a	Involved in the study
☐	☒ Antibodies
☐	☒ Eukaryotic cell lines
☒	☐ Palaeontology and archaeology
☐	☒ Animals and other organisms
☐	☒ Human research participants
☒	☐ Clinical data
☒	☐ Dual use research of concern

Methods

n/a	Involved in the study
☒	☐ ChIP-seq
☐	☒ Flow cytometry
☒	☐ MRI-based neuroimaging

Antibodies

Antibodies used

I. Western Blot - Primary Antibodies

1. 4E-BP1; Source: Rabbit; Dilution: 1:1,000; Product Number: 9452; Company: Cell Signaling Technology
2. BiP (C50B12); Source: Rabbit; Dilution: 1:500 - 1:1,000; Product Number: 3177; Company: Cell Signaling Technology
3. Cleaved PARP (Asp214; 7C9); Source: Mouse; Dilution: 1:500 - 1:1,000; Product Number: 9548; Company: Cell Signaling Technology
4. c-Myc (D84C12); Source: Rabbit; Dilution: 1:1,000; Product Number: 5605; Company: Cell Signaling Technology
5. GAPDH (14C10); Source: Rabbit; Dilution: 1:1,000; Product Number: 2118; Company: Cell Signaling Technology
6. LC3B; Source: Rabbit; Dilution: 1:1,000; Product Number: ab48394; Company: Abcam
7. MKK3 (D4C3); Source: Rabbit; Dilution: 1:1,000; Product Number: 8535; Company: Cell Signaling Technology
8. MKK6 (D31D1); Source: Rabbit; Dilution: 1:1,000; Product Number: 8550; Company: Cell Signaling Technology
9. p38 MAPK (D13E1); Source: Rabbit; Dilution: 1:500 - 1:1,000; Product Number: 8690; Company: Cell Signaling Technology
10. p38 MAPK (M138); Source: Mouse; Dilution: 1:1,000; Product Number: ab31828; Company: Abcam
11. p70 S6-kinase (E8K6T); Source: Rabbit; Dilution: 1:1,000; Product Number: 9202; Company: Cell Signaling Technology
12. Phospho-4E-BP1 (Thr37/46); Source: Rabbit; Dilution: 1:1,000; Product Number: 9459; Company: Cell Signaling Technology
13. Phospho-MKK3(Ser189)/MKK6 (Ser207) (D8E9); Source: Rabbit; Dilution: 1:1,000; Product Number: 12280; Company: Cell Signaling Technology
14. Phospho-MLK3 (Thr277, Ser281); Source: Rabbit; Dilution: 1:500 - 1:1,000; Product Number: ab191530; Company: Abcam
15. Phospho-p38 MAPK (Thr180/Tyr182); Source: Rabbit; Dilution: 1:500 - 1:1,000; Product Number: 9211; Company: Cell Signaling Technology
16. Phospho-p70 S6-kinase (Thr389; 108D2); Source: Rabbit; Dilution: 1:1,000; Product Number: 9234; Company: Cell Signaling Technology
17. Phospho-S6 Ribosomal Protein (Ser235/236; 2F9); Source: Rabbit; Dilution: 1:1,000; Product Number: 4856; Company: Cell Signaling Technology
18. Phospho-SAPK/JNK (Thr183/Tyr185; 81E11); Source: Rabbit; Dilution: 1:1,000; Product Number: 4668; Company: Cell Signaling Technology
19. Phospho-SEK1/MKK4 (Ser257; C36C11); Source: Rabbit; Dilution: 1:1,000; Product Number: 4514; Company: Cell Signaling Technology
20. Phospho-Ulk1 (Ser757); Source: Rabbit; Dilution: 1:1,000; Product Number: 6888; Company: Cell Signaling Technology
21. S6 Ribosomal Protein (5G10); Source: Rabbit; Dilution: 1:1,000; Product Number: 2217; Company: Cell Signaling Technology
22. SAPK/JNK; Source: Rabbit; Dilution: 1:1,000; Product Number: 9252; Company: Cell Signaling Technology
23. SCD1 (M38); Source: Rabbit; Dilution: 1:1,000; Product Number: 2438; Company: Cell Signaling Technology
24. Ulk1 (EPR4885(2)); Source: Rabbit; Dilution: 1:1,000; Product Number: ab128859; Company: Abcam
25. β-actin (13E5); Source: Rabbit; Dilution: 1:1,000; Product Number: 4970; Company: Cell Signaling Technology
26. β-actin (8H10D10); Source: Mouse; Dilution: 1:1,000; Product Number: 3700; Company: Cell Signaling Technology
27. β-actin; Source: Rabbit; Dilution: 1:10,000; Product Number: A2066; Company: Sigma-Aldrich

II. Western Blot - Secondary Antibodies

1. DyLight™ 680 IgG (H+L) Cross-Adsorbed Goat anti-Mouse; Source: Goat; Dilution: 1:10,000; Product Number: 35519; Company: Thermo Fisher Scientific
2. DyLight™ 680 IgG (H+L) Cross-Adsorbed Goat anti-Rabbit; Source: Goat; Dilution: 1:10,000; Product Number: 35569; Company: Thermo Fisher Scientific
3. DyLight™ 800 IgG (H+L) Cross-Adsorbed Goat anti-Rabbit; Source: Goat; Dilution: 1:10,000; Product Number: SA510036; Company: Thermo Fisher Scientific
4. IRDye® 680LT Goat anti-Mouse IgG Secondary Antibody; Source: Goat; Dilution: 1:80,000; Product Number: 926-68020; Company: LI-COR Biosciences

5. IRDye® 680LT Goat anti-Rabbit IgG Secondary Antibody; Source: Goat; Dilution: 1:80,000; Product Number: 926-68021; Company: LI-COR Biosciences
6. IRDye® 800CW Goat anti-Mouse IgG Secondary Antibody; Source: Goat; Dilution: 1:10,000; Product Number: 926-32210; Company: LI-COR Biosciences
7. IRDye® 800CW Goat anti-Rabbit IgG Secondary Antibody; Source: Goat; Dilution: 1:10,000; Product Number: 926-32211; Company: LI-COR Biosciences

III. Immunofluorescence microscopy

1. Alexa Fluor 555 goat anti-mouse IgG; Source: Goat; Dilution: 1:1,000; Product Number: A32727; Company: Thermo Fisher Scientific
2. Anti-GRP78 (A-10); Source: Mouse; Dilution: 1:250; Product Number: sc-376768; Company: Santa Cruz Biotechnology

IV. Cell sorting

1. APC conjugated anti-mouse c-Kit (clone 2B8); Source: Rat; Dilution: 1:100; Product Number: 17-1171; Company: Thermo Fisher Scientific
2. PE/Cy7 anti-mouse Sca-1 (clone E13-161.7); Source: Rat; Dilution: 1:100; Product Number: 122513; Company: BioLegend

V. Bead-based cell isolation

1. Anti-IgM (clone R6-60.2); Source: Rat; Dilution: 1:100; Product Number: 553406; Company: BD Biosciences

VI. Flow cytometry

1. Anti-CD19-APC (clone 1D3); Source: Rat; Dilution: 1:100; Product Number: 152410; Company: BioLegend
2. Anti-IgM-PE-Cy7 (clone eB121-15F9); Source: Rat; Dilution: 1:100; Product Number: 25-5890-82; Company: Thermo Fisher Scientific

Validation

All antibodies used in the current study were validated by the manufacturers. Detailed information about the manufacturer's validation process can be found at:

1) Abcam: https://www.abcam.com/primary-antibodies/how-we-validate-our-antibodies
2) BD Biosciences: https://www.bdbiosciences.com/en-us/products/reagents/flow-cytometry-reagents/research-reagents
3) BioLegend: https://www.biolegend.com/en-us/quality/product-development
4) Cell Signaling Technology: https://www.cellsignal.com/about-us/cst-antibody-validation-principles
5) LI-COR Biosciences: https://www.licor.com/bio/reagents/irdye-infrared-dyes
6) Santa Cruz Biotechnology: https://www.scbt.com/about-us
7) Sigma-Aldrich: https://www.sigmaaldrich.com/US/en/products/protein-biology/antibodies/enhanced-validation-ab
8) Thermo Fisher Scientific: https://www.thermofisher.com/at/en/home/life-science/antibodies.html

The following list gives the species reactivity of each antibody according to manufacturer's information:

I. Western Blot - Primary Antibodies

1. 4E-BP1; Source: Rabbit; Species Reactivity: Human, Mouse, Rat, Monkey
2. BiP (C50B12); Source: Rabbit; Species Reactivity: Human, Mouse
3. Cleaved PARP (Asp214; 7C9); Source: Mouse; Species Reactivity: Mouse
4. c-Myc (D84C12); Source: Rabbit; Species Reactivity: Human, Mouse, Rat
5. GAPDH (14C10); Source: Rabbit; Species Reactivity: Human, Mouse, Rat, Monkey, Bovine, Pig
6. LC3B; Source: Rabbit; Species Reactivity: Mouse, Rat, Human
7. MKK3 (D4C3); Source: Rabbit; Species Reactivity: Human, Mouse, Rat, Monkey
8. MKK6 (D31D1); Source: Rabbit; Species Reactivity: Human, Mouse, Rat, Monkey
9. p38 MAPK (D13E1); Source: Rabbit; Species Reactivity: Human, Mouse, Rat, Hamster, Monkey, Bovine, Pig
10. p38 MAPK (M138); Source: Mouse; Species Reactivity: Mouse, Rat, Cow, Dog, Human, African green monkey, Syrian hamster
11. p70 S6-kinase (E8K6T); Source: Rabbit; Species Reactivity: Human, Mouse, Rat, Monkey
12. Phospho-4E-BP1 (Thr37/46); Source: Rabbit; Species Reactivity: Human, Mouse, Rat, Monkey
13. Phospho-MKK3(Ser189)/MKK6 (Ser207) (D8E9); Source: Rabbit; Species Reactivity: Human, Mouse, Rat, Monkey
14. Phospho-MLK3 (Thr277, Ser281); Source: Rabbit; Species Reactivity: Mouse, Rat, Human
15. Phospho-p38 MAPK (Thr180/Tyr182); Source: Rabbit; Species Reactivity: Human, Mouse, Rat, Monkey, D. melanogaster, Pig, S. cerevisiae
16. Phospho-p70 S6-kinase (Thr389; 108D2); Source: Rabbit; Species Reactivity: Human, Mouse, Rat, Monkey
17. Phospho-S6 Ribosomal Protein (Ser235/236; 2F9); Source: Rabbit; Species Reactivity: Human, Mouse, Rat, Monkey
18. Phospho-SAPK/JNK (Thr183/Tyr185; 81E11); Source: Rabbit; Species Reactivity: Human, Mouse, Rat, D. melanogaster, S. cerevisiae
19. Phospho-SEK1/MKK4 (Ser257; C36C11); Source: Rabbit; Species Reactivity: Human, Mouse, Rat, Monkey
20. Phospho-Ulk1 (Ser757); Source: Rabbit; Species Reactivity: Human, Mouse, Monkey
21. S6 Ribosomal Protein (5G10); Source: Rabbit; Species Reactivity: Human, Mouse, Rat, Monkey
22. SAPK/JNK; Source: Rabbit; Species Reactivity: Human, Mouse, Rat, Hamster, Monkey, Zebrafish, Bovine, S. cerevisiae
23. SCD1 (M38); Source: Rabbit; Species Reactivity: Human, Mouse

24. Ulk1 (EPR4885(2)); Source: Rabbit; Species Reactivity: Mouse, Rat, Human
25. β-actin (13E5); Source: Rabbit; Species Reactivity: Human, Mouse, Rat, Monkey, Bovine, Pig
26. β-actin (8H10D10); Source: Mouse; Species Reactivity: Human, Mouse, Rat, Hamster, Monkey, Dog
27. β-actin; Source: Rabbit; Species Reactivity: Wide range, Human, Chicken, Amoeba, Slime mold, Vertebrates

II. Western Blot - Secondary Antibodies

1. DyLight™ 680 IgG (H+L) Cross-Adsorbed Goat anti-Mouse; Source: Goat; Species Reactivity: Mouse
2. DyLight™ 680 IgG (H+L) Cross-Adsorbed Goat anti-Rabbit; Source: Goat; Species Reactivity: Rabbit
3. DyLight™ 800 IgG (H+L) Cross-Adsorbed Goat anti-Rabbit; Source: Goat; Species Reactivity: Rabbit
4. IRDye® 680LT Goat anti-Mouse IgG Secondary Antibody; Source: Goat; Species Reactivity: Mouse
5. IRDye® 680LT Goat anti-Rabbit IgG Secondary Antibody; Source: Goat; Species Reactivity: Rabbit
6. IRDye® 800CW Goat anti-Mouse IgG Secondary Antibody; Source: Goat; Species Reactivity: Mouse
7. IRDye® 800CW Goat anti-Rabbit IgG Secondary Antibody; Source: Goat; Species Reactivity: Rabbit

III. Immunofluorescence microscopy

1. Alexa Fluor 555 goat anti-mouse IgG; Source: Goat; Species Reactivity: Mouse
2. Anti-GRP78 (A-10); Source: Mouse; Species Reactivity: Mouse, Rat, Human, Canine, Bovine, Porcine, Avian

IV. Cell sorting

1. APC conjugated anti-mouse c-Kit (clone 2B8); Source: Rat; Species Reactivity: Mouse, Pig
2. PE/Cy7 anti-mouse Sca-1 (clone E13-161.7); Source: Rat; Species Reactivity: Mouse
V. Bead-based cell isolation

1. Anti-IgM (clone R6-60.2); Source: Rat; Species Reactivity: Mouse

VI. Flow cytometry

1. Anti-CD19-APC (clone 1D3); Source: Rat; Species Reactivity: Mouse
2. Anti-IgM-PE-Cy7 (clone eB121-15F9); Source: Rat; Species Reactivity: Mouse

Eukaryotic cell lines

Policy information about cell lines

Cell line source(s)	Cell lines were from the DSMZ-German Collection of Microorganisms and Cell Cultures (DSMZ, Braunschweig, Germany), the American Type Culture Collection (ATCC, Manassas, VA) or the Japanese Collection of Research Bioresources Cell Bank (JCRB Cell Bank, Ibaraki, Japan).<br><br>1. NIH-3T3, # ACC 59, DSMZ<br>2. HeLa, # ACC 57, DSMZ<br>3. HT29, # HTB-38, ATCC<br>4. HEK-293, # CRL-1573, ATCC<br>5. HepG2, # ACC 180, DSMZ<br>6. MCF-7, # HTB-22, ATCC<br>7. HUH-7, # JCRB0403, JCRB Cell Bank<br>8. MM-6, # ACC 124, DSMZ<br>9. HUVECs were isolated from human umbilical cord veins and kindly provided by Dr. Alexander Mosig (University Hospital Jena, Germany)
Authentication	The identity of MCF-7 and HEK-293 cells was confirmed. The authentication was performed by Multiplexion (Friedrichshafen, Germany; December, 2020) using Single Nucleotide Polymorphism (SNP) profiling (Multiplex Cell Line Authentication, https://www.multiplexion.de/en/cell-line-testing-service/multiplex-human-cell-line-authentication). DNA for SNP profiling was isolated from cell pellets using an innuPREP DNA Mini Kit (Analytik Jena) according to the manufacturer's instructions. Other cell lines were not authenticated. Cell morphology of all cell lines was regularly inspected.
Mycoplasma contamination	Cell lines were tested for mycoplasma and found negative for contamination.
Commonly misidentified lines (See ICLAC register)	HEK-293 is described as misidentified cell line, which we studied because of the high basal p38 MAPK phosphorylation.

Animals and other organisms

Policy information about studies involving animals; ARRIVE guidelines recommended for reporting animal research

Laboratory animals	1) Old male and female C57BL/6JRj mice (26 month); 2) Young male and female C57BL/6JRj mice (6-8 month); 3) Male and female young (3 to 6 month) and old (18 to 24 month) C57BL/6 mice; 4) Male 8-week-old wildtype and Tg(IghMyc)22Bri ("Eμ-Myc") mice with C57BL/6JRj background and male and female tumor-bearing Eμ-Myc transgenic mice of 15 to 52 weeks of age; 5) Male C57BL/6

	mice (4 weeks); 6) Schmidtea mediterranea asexual biotype
Wild animals	The study did not involve wild animals.
Field-collected samples	The study did not involve samples collected from the field.
Ethics oversight	Ethical commission of the Friedrich-Schiller University Jena and the University Hospital Jena

Note that full information on the approval of the study protocol must also be provided in the manuscript.

Human research participants

Policy information about studies involving human research participants

Population characteristics	Human leukocyte concentrates were provided by the Institute for Transfusion Medicine of the University Hospital Jena (Germany). These subjects donated blood every 8 to 12 weeks, had no apparent infections, inflammatory conditions, or current allergic reactions (according to prior physical inspection by a clinician) and had not taken antibiotics or anti-inflammatory drugs for at least 10 days prior to blood collection.
Recruitment	Venous blood was collected from fasted (12 h) adult (18–65 years) male and female registered healthy volunteers, with informed consent, by the Institute for Transfusion Medicine of the University Hospital Jena (Germany). Authors of this study were neither involved in the recruitment nor selection of blood donors, who were randomly assigned by the Transfusion Medicine to research requests.
Ethics oversight	Ethical commission of the Friedrich-Schiller-University Jena

Note that full information on the approval of the study protocol must also be provided in the manuscript.

Flow Cytometry

Plots

Confirm that:

- [x] The axis labels state the marker and fluorochrome used (e.g. CD4-FITC).
- [x] The axis scales are clearly visible. Include numbers along axes only for bottom left plot of group (a 'group' is an analysis of identical markers).
- [x] All plots are contour plots with outliers or pseudocolor plots.
- [x] A numerical value for number of cells or percentage (with statistics) is provided.

Methodology

Sample preparation	1) NIH-3T3 fibroblasts were harvested and stained with propidium iodide and annexin-V using either an Annexin V Apoptosis Detection Kit APC (Thermo Fisher Scientific) or an Annexin V Apoptosis Detection Kit FITC (Thermo Fisher Scientific) according to the manufacturer's instructions.<br>2) To obtain hematopoetic stem and progenitor cells, bone marrow cells, which were freshly isolated from tibia and femur of mice, were enriched by magnetic activated cell separation, immunolabeled with Sca-1 and lineage antibodies and sorted by FACS.
Instrument	BD LSR Fortessa flow cytometer (BD Biosciences); FACSAria II instrument (BD Biosciences, Franklin Lakes, NJ)
Software	BD FACSDiva 8.0.1 (BD Biosciences), Flowlogic V7 software (Miltenyi Biotech); FlowJo v10 software (BD Biosciences)
Cell population abundance	Flow cytometric studies were conducted with a cell line (mouse NIH-3T3 cells). Hematopoetic stem and progenitor cells were selected as Lin- cKit+ ScaI+ (KSL) cells and Lin- cKit+ ScaI- myeloid progenitor (MP) cells.
Gating strategy	Cells were pre-gated on FSC/SSC following single cell identification to exclude debris and aggregates. Pre-gated cells were analyzed for Annexin V and PI signals. Boundaries between positive and negative fractions were defined using single stainings of treated samples.

- [x] Tick this box to confirm that a figure exemplifying the gating strategy is provided in the Supplementary Information.
